# The influence of resilience-based management on coral reef monitoring: A systematic review

**DOI:** 10.1371/journal.pone.0172064

**Published:** 2017-02-10

**Authors:** Vivian Y. Y. Lam, Christopher Doropoulos, Peter J. Mumby

**Affiliations:** 1 Marine Spatial Ecology Lab, School of Biological Sciences, The University of Queensland, St. Lucia, Queensland, Australia; 2 CSIRO Oceans and Atmosphere, Dutton Park, Queensland, Australia; Academia Sinica, TAIWAN

## Abstract

With rapid changes taking place on coral reefs, managers and scientists are faced with prioritising interventions that might avoid undesirable losses in ecosystem health. The property of resilience captures how reefs react and respond to stressors and environmental changes. Therefore, in principle, management goals are more likely to be realised if resilience theory is used to inform decision making and help set realistic expectations for reef outcomes. Indeed, a new approach to reef management has been termed ‘resilience-based management’ (RBM). Yet, resilience concepts have often been criticised for being vague, difficult to operationalise, and beset by multiple definitions. Here, we evaluate how the advent of RBM has changed one aspect of reef management: assessment and monitoring. We compare the metrics used in conventional monitoring programs with those developed through resilience assessments and find that the latter have a stronger focus on ecological processes and exposure to environmental drivers. In contrast, monitoring tends to focus on metrics of reef state and has greater taxonomic resolution, which provides comprehensive information on the nature of changes but does not predict the future responses of reefs in part because it is difficult to extrapolate statistical trends of complex ecological systems. In addition, metrics measured by resilience studies are more diverse, owing in part to the reliance of state metrics as proxies of processes given the difficulty in quantifying key ecological processes directly. We conclude by describing practical ways of improving resilience assessments, and avenues for future research.

## Introduction

The accelerating rate of change in many ecosystems [1–5] and apparent lack of recovery [6–8] has led to the perception that it is more appropriate to manage for ecosystem resilience rather than ecosystem state and condition [9–11]. This shift in management application has been termed ‘resilience-based management’ (RBM), which attempts to manage the resilience of systems explicitly. Resilience was first described by Holling [12] as a dynamical property of ecosystems such that they continue to gravitate toward one particular state versus another even when subjected to perturbations. A resilient coral reef, for example, will tend to exhibit recovery towards a coral dominant state even if subsequent disturbances prevent corals from eventually dominating [13]. RBM steers management actions towards the preservation of fundamental ecosystem functions, structure, identity and feedbacks [9]. RBM departs from the classic view of steady-state resource management and instead attempts to focus on the processes that govern system dynamics [14]. Contrary to the emphasis on the maintenance of a static perceived optimal state in traditional management approaches, RBM is closely tied to the prevention of regime shifts, whereby a conspicuous change to the structure and function of a system occurs once a threshold is surpassed [15, 16]. Regime shifts involve complex feedback mechanisms that affect system dynamics, hence, a critical aspect of managing for resilience is a thorough understanding of ecological processes of the relevant ecosystems.

Monitoring and assessment are integral components of ecosystem management [17, 18], providing key information through empirical measurements and trend identification [19]. Historically, monitoring activities deliver a detailed account on ecosystem states with an emphasis on the abundance of important biological species. However, state indicators are not able to reflect important underlying system, for example nonlinear interactions and feedback loops such as those between corals, algae and herbivores [20–22]. What is observed (i.e. the state) is the result of multiple interactions among the biophysical components of an ecosystem. Thus, in addition to ecosystem state changes, monitoring of ecological processes is fundamental to the successful implementation of RBM [23–25]. Ecological processes maintain the functioning of an ecosystem and are often responsible for the dynamics of a system, including processes such as energy flow, nutrient cycling and disturbance regimes [26]. In the context of this study, ecological processes are defined as components that affect reef ecosystem functions, such as recruitment and connectivity [27].

There is a long history of coral reef monitoring in different parts of the world [28, 29], with the majority of international programs initiated in the mid-90s [30]. The aim of these programs is to monitor and reflect the health of reef ecosystems, implemented through comprehensive surveys that record changes in the abundance of organisms and how they react to impacts [31]. Conversely, metrics selected for resilience assessments aim to measure processes [32], yet this is often difficult to achieve because many dynamic processes are not easily observable [33], such as larval supply, settlement behaviour and post-settlement mortality.

Quantification is a crucial facet in the operationalisation of RBM, and efforts have concentrated on the identification of suitable resilience metrics [34–37]. Although the focus on system dynamics for resilience assessments is apparent [38], the extent to which RBM has transformed how reefs are evaluated and monitored is unclear. This study investigates how the introduction of RBM has changed the way reefs are surveyed. Specifically, we examine whether RBM has translated into an explicit change in the metrics surveyed and how it differs from earlier management approaches. To do so, metrics utilised by monitoring programs are compared to those adopted by more recent RBM approaches. Based on our results, new directions for resilience science are identified, in particular, on operationalising the metrics used for management and maximising the value of historical monitoring data.

## Methods

**Fig 1:**
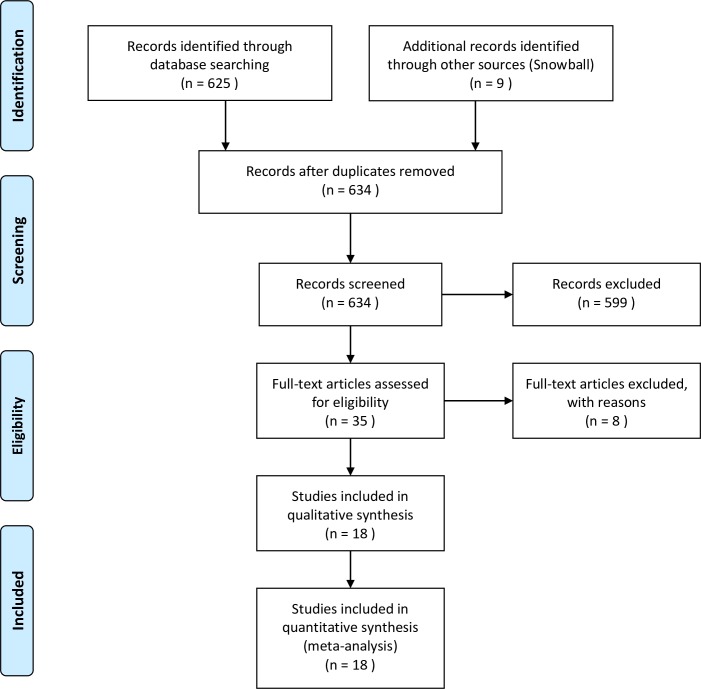
PRISMA flow diagram for literature search.

A great challenge is to identify and keep track of emerging resilience approaches and papers, hence a literature review was conducted to identify coral reef resilience studies that documented quantifiable metrics. A broad list of search terms was used to avoid missing any relevant research because resilience and monitoring studies often use different terminology. The search was conducted in July 2016 using the Web of Science service (ISI Thomson Reuters), with the key topics of “reef” and “resilience/regime shift” and “driver*/assess*/measure*/variable*/indicator*” and no limits on publication dates, which resulted in a total of 625 studies. A large proportion (95.8%) of the resulting studies included conceptual and theoretical research, and further analysis resulted in 10 resilience studies that contained quantifiable metrics suitable for analysis. Studies that only focused on specific groups of reef organisms were not included, for example fish functional groups in Green et al. [39]. Case studies using the same protocols were not included (total of 4 studies). For example, where Ladd et al. [40] applied the resilience index developed by Maynard et al. [36] in Mexico. Following, a snowball sampling technique [41] was employed that expanded the search using reference lists from the initial pool of studies. Monitoring protocols were taken from well-established long-term monitoring programs with widely-accepted monitoring procedures. Finally, twelve studies and six internationally recognised coral reef monitoring programs were identified that contained published protocols to represent resilience assessments and monitoring programs respectively. The classification of groups was based on the goals stated in each study, with the rationale behind the groupings outlined in S1 Table. A brief description of the data collection can be found in the PRISMA flow diagram (Fig 1) and checklist (S1 Checklist).

The initial stage of analysis included a thorough examination and documentation of metrics listed in each study, and the reasons for monitoring noted where applicable (S1 Table). Minor adjustments were made to the original terms describing metrics to minimise any biases resulting from heuristics, giving a final list of 136 standard metrics from both resilience studies and monitoring programs (S2 Table). The 136 standard metrics were then used to populate the data matrix, with a binomial response (present/absent) applied to each metric for each study in a multivariate data frame. Studies were visualised using non-metric multidimensional scaling (nMDS) and analysed for statistical significance among groups (resilience assessment, monitoring program) and locations (Caribbean, Pacific, Indian Ocean and global assessments) using PERMANOVA with 999 permutations to generate P values. Multivariate homogeneity of variance was tested using PERMDISP. SIMPER analysis was conducted to identify the key metrics that contribute to the similarities and differences between and within significant factors. Analyses were based on a Jaccard dissimilarity matrix, appropriate for presence-absence data [42], and conducted using the software Primer-E v6 [43] with the PERMANOVA extension.

The second analysis classified the full list of 136 standard metrics into 28 higher-level metrics (S2 Table), grouped into the general categories of biological community, disturbances, ecological processes and site characteristics for comparative analysis (Table 1). Abundance and diversity metrics were grouped under ‘biological community’, and include algae, corals, fish and other organisms found in the benthic community. Disturbance metrics that impacted the reef were grouped under ‘disturbances’, broadly separated into anthropogenic, biological, physical and physiological (Table 1). Process-oriented metrics were grouped under ‘ecological processes’, and include critical coral processes such as growth, recruitment and connectivity. Finally, metrics that reflected reef conditions were grouped under ‘site characteristics’, and contain information on the location, management status and general environment of sites surveyed. Metrics were converted into presence/absence data for each study and the proportion of studies that measured the 28 higher-level metrics was calculated to indicate metric prevalence within the two groups (resilience assessment, monitoring program). Metric prevalence is used here to indicate emphasis on certain metrics by resilience assessments and monitoring programs respectively.

**Table 1:** Higher-level metrics classified under four general categories.

Category	Higher-level Metric	General areas covered
**Biological community**	1. Algal cover	
(Richness, abundance,	2. Algal biomass	
size, composition)	3. Algal height	
	4. Coral community	Size, diversity, growth form
	5. Coral cover	
	6. Fish abundance	
	7. Fish size	
	8. Fish biomass	
	9. Fish community	Diversity
	10. Organisms (harmful)	
	11. Organisms (unharmful)	
	12. Other invertebrates	Sponge, gorgonian, tunicate
	13. Seagrass	
**Disturbances**	14. Anthropogenic	Development
		Human population
		Nutrients
		Water quality
	15. Biological	Bioerosion
		Invasive species
		Predation
	16. Physical	Destructive fishing Hurricane/cyclone
		Physical impact
	17. Physiological	Bleaching/thermal regime
		Disease
**Ecological processes**	18. Connectivity	
	19. Competition	
	20. Growth	
	21. Herbivory	
	22. Mortality	
	23. Recruitment	
	24. Reproduction	
**Site characteristics**	25. Location & geomorphology	Depth
		Habitat
		Habitat complexity
		Location
		Reef type
		Reef zone
		Slope
	26. Physical	Light conditions
		Temperature/thermal regime
		Salinity
		Wave exposure/tides/mixing
		Turbidity/visibility
	27. Substrate	Rock, rubble, sand, silt, suitable for recruitment (substrate availability)
	28. Management Status	Management

Classification used in Fig 3. For categorisation of original variables, please refer to S2 Table.

A final analysis was conducted to elucidate the potential of different metrics to provide information on reef processes used in the two groups of studies. Metrics from each study were classified into four broad categories: 1) state, 2) state/proxy, 3) proxy; and 4) process (S2 Table). ‘State’ represents static metrics (e.g., abundance), whereas metrics that directly measure rates and ecological processes are grouped under ‘process’ (e.g. recruitment, growth). Various state metrics are often used as proxies for ecological processes, such as herbivore biomass for herbivory, and these are grouped under ‘state/proxy’. Finally, metrics that were used directly as a proxy for a process within a study (i.e. indices developed to represent processes and disturbances such as coral submersion to estimate stress to corals when exposed to air, or number of boats to represent human impacts) were classified under ‘proxy’. When a state-only metric was explicitly used as a proxy for a process (42 out of 229 cases), metrics typically classified as ‘state’ (e.g., coral cover) were also grouped under the category of ‘state/proxy’. Metrics were considered on a case by case basis depending on how they were used in the particular study and the underlying measurement rationale (S2 Table). The proportion of metrics classified into each category were averaged to indicate differences between resilience assessments and monitoring programs.

## Results

**Fig 2:**
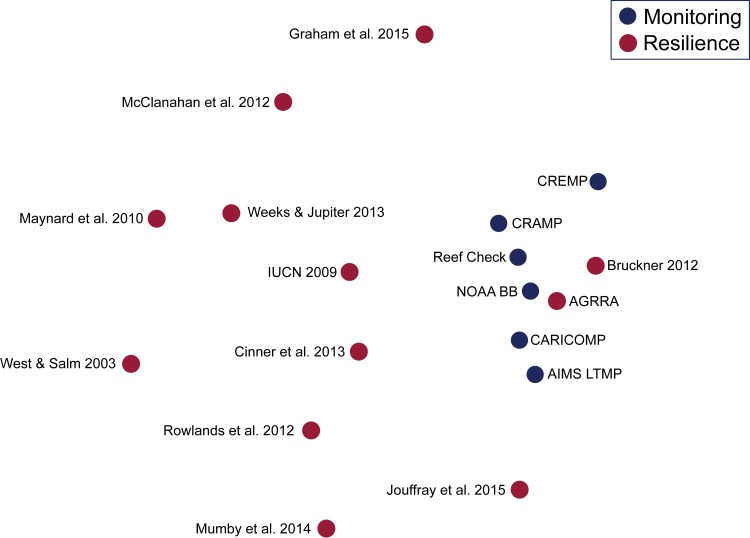
Non-metric multidimensional scaling plot (nMDS) comparing metrics used by studies with monitoring and resilience objectives. AIMS = Long Term Monitoring Program, Australia Institute of Marine Science; AGGRA = Atlantic and Gulf Rapid Reef Assessment; CARICOMP = Caribbean Coastal Marine Productivity; CRAMP = Hawai‘i Coral Reef Assessment and Monitoring Program; CREMP = Coral Reef Evaluation and Monitoring Project by Florida Fish and Wildlife Conservation Commission; IUCN = International Union for Conservation of Nature; NOAA BB = National Oceanic and Atmospheric Administration, Center for Coastal Monitoring and Assessment, Biogeography Branch.

A total of 136 standard coral reef metrics were recorded, with monitoring programs and resilience assessments totalling 61 and 126 metrics respectively (S2 Table). Metrics used for resilience assessments differed significantly from those employed for monitoring (p = 0.01, S3A Table; Fig 2), with an average dissimilarity of 75%. Despite the high dissimilarity between the two groups, metrics from resilience assessments encompassed many aspects observed by monitoring programs and had a higher multivariate dispersion (PERMDISP average dispersion: resilience = 59.1; monitoring = 47.1, S3B Table), implying that resilience assessments measure a much wider range of metrics compared to monitoring programs. Two resilience studies in particular [44, 45], showed more similarity to monitoring studies than the other resilience assessments (Fig 2), and this may reflect their transitional nature. For example, AGRRA [44] developed from monitoring methods in the late 1990s, but there was an explicit intent to include process-level metrics, such as densities of juvenile corals and rates of herbivory (Mumby, pers. obs.). The influence of geographical location on measured metrics and the interaction between the two groups were both non-significant (Location: p = 0.18; Group x Location: p = 0.534; S3A Table).

**Fig 3:**
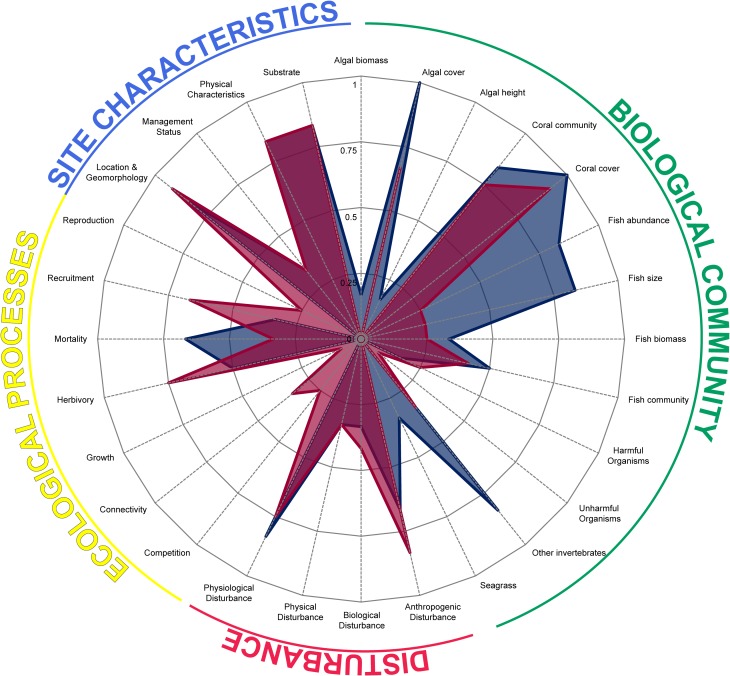
Higher-level metrics used by monitoring and resilience studies. Blue and red areas represent monitoring programs and resilience assessments respectively. Axes points represent the proportion of studies within each study group that measured a given metric. Refer to S2 Table for original metrics categorised under each higher-level metric.

Monitoring studies had an average similarity of 42%. Key benthic state metrics such as coral cover (living, diseased, bleached and dead), algal cover (macroalgae, turf, crustose coralline algae), sessile invertebrate cover (soft coral, gorgonian, sponge, anemones, tunicates, ascidians) and substrate were used in more than two-thirds of the monitoring programs. Metrics reflecting biodiversity (coral and fish diversity) and site characteristics (location, habitat, temperature, depth, rugosity and turbidity) were also found in over half of the studies (Fig 3, S4 Table). Metrics used in resilience assessments were more numerous and diverse, with a much lower average within-group similarity of 22%. Despite the high diversity of metrics being recorded, benthic components such as coral, macroalgal and substrate cover were used in more than half of the resilience assessments alongside drivers such as thermal regime, disease, fishing pressure and depth (Fig 3, S4 Table).

Evidently, resilience assessments tend to focus on ecological and environmental processes, whereas monitoring programs concentrate on the biological community including benthic cover and organism abundances (Fig 3). Resilience studies tend to target specific functions, such as converting sea surface temperatures into thermal regime indices and identifying substrate categories suitable for coral recruitment (i.e. substrate availability). Monitoring programs tend to record sea surface temperatures changes and document all substrate covers (i.e. sand, rubble and rock), leaving the analyst to determine the functions of the categories. Also, monitoring programs do not attempt to measure complex processes such as connectivity, competition and reproduction whereas most resilience assessments at least seek proxies for such processes. Monitoring studies often took note of other living organisms such as sessile and motile invertebrates, whereas resilience assessments focused on specific categories that are used as proxies for reef processes.

**Fig 4:**
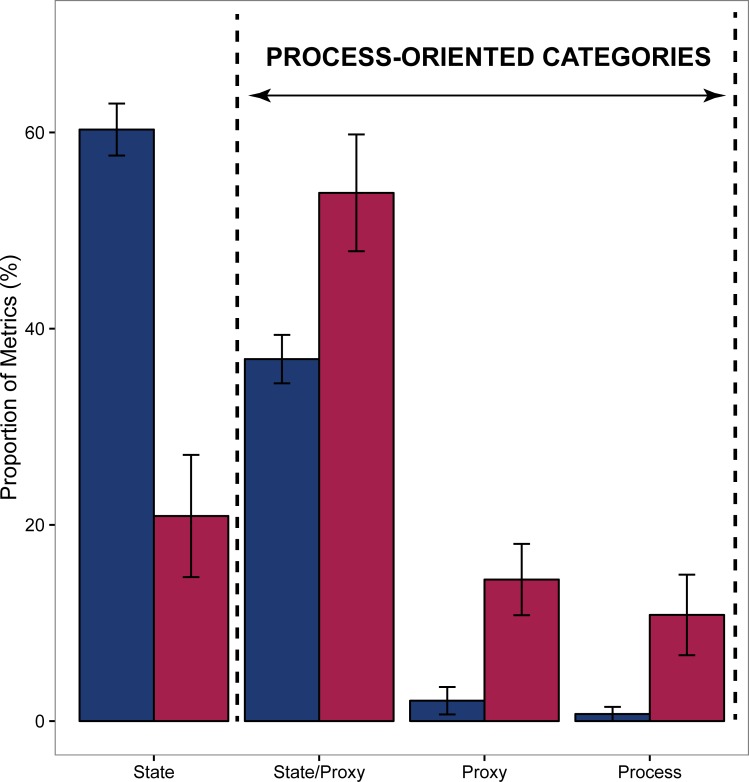
The potential of metrics to provide information on processes used in monitoring and resilience studies. Blue and red bars represent monitoring and resilience assessments respectively. Error bars represent standard errors.

A more detailed examination of measurements by monitoring programs revealed that up to 35% metrics are state variables that can also be used proxies for processes (Fig 4), implying that scope exists to interpret monitoring data from a resilience perspective. The majority of metrics from monitoring programs are state variables (60%), with a strong focus on counts, abundances and diversity. In contrast, ~80% of the resilience assessment metrics fall under state/proxy, proxy and process categories. In resilience assessments, over half of the total measurements were states used as proxies for processes, signifying the critical role of surrogates in the development of RBM.

## Discussion

### Monitoring and resilience: “states” vs. “rates”

We found substantial differences in the metrics utilised by monitoring programs and resilience assessments and confirm that the introduction of RBM has influenced how reef surveys are conducted. Our study provides clear evidence that the intent of RBM has translated into a real change in metrics surveyed, documenting a shift towards more process-oriented measurements as suggested by conceptual advice [46, 47]. The different foci of monitoring and resilience assessments reflect their respective objectives. Monitoring programs are concerned with the detection of detailed changes in the reef community, relying on state metrics that can be easily quantified. A strong emphasis on the biological community led to a higher degree of similarity within the monitoring group. Resilience assessments, on the other hand, have evolved from basic monitoring protocols to include additional ecological and environmental factors that provide insight on process dynamics [45, 48]. Hence, resilience assessments often incorporate many of the metrics used in monitoring assessments, as shown in AGRRA [44] and Bruckner [45], as well as a larger variety of parameters. Of interest, the National Coral Reef Monitoring Program (NCRMP), initiated in recent years to develop a nationally coordinated and consistent monitoring program, also illustrates the move from a traditional monitoring program to a program that focuses on ecological processes. In addition to critical state metrics that have traditionally been monitored by NOAA, NCRMP also promotes additional indicators that contribute to coral reef ecosystem function such as growth and bioerosion rates, reproduction and recruitment and hydrodynamics [49].

Resilience assessments have a much higher variability of metrics used among studies than do monitoring programs. Results from the PERMDISP analysis show that multivariate dispersion observed for resilience assessments was 1.3–times more than monitoring programs, and there are a number of reasons for this higher variability. First, resilience metrics include variables scored using systems such as the Likert scale to assign values rapidly in the field that would otherwise be complicated to quantify (e.g. self-, local- and distant-seeding that contributes to connectivity [35]), whereas monitoring programs do not attempt to quantify such variables. Second, metric variability within the resilience group occurs with the inclusion of modelling studies that incorporate the direct quantification of processes and rates such as growth, mortality and recruitment, which is hard to achieve in field-based resilience assessments, especially for snapshot surveys [50, 51]. Third, monitoring studies have well-established protocols whereas resilience metrics are often modified and adapted to study sites or region [36, 52].

In line with literature advocating for the development of surrogates for processes measured in resilience assessments [33], it is encouraging that resilience studies utilised a high proportion of state metrics, with the intent of using them as proxies for reef processes. Evidently, state metrics, when used appropriately, can provide valuable information on ecosystem dynamics [53]. However, there are certain advantages and disadvantages associated with the use of proxies. Multiple proxies can be used to represent the same process or disturbance, and allows resilience assessments to be more flexible in data acquisition. For instance, distance to the nearest river mouth or human population density are both used as proxies for human disturbance [54]. However, proxies also contribute to the high diversity of metrics used in resilience assessments. High metric variability may add to the complexities of quantifying resilience using field-based measurements [55, 56], thus, further work is needed to identify a uniform set of resilience metrics. Particular emphasis should be given to improving metrics for processes and drivers, as many of these are hard to observe using single proxies, making unification difficult [57].

### From metrics to management

Despite finding clear differences between the metrics used in monitoring programs and resilience assessments, a substantial proportion of monitoring metrics have the potential to be used as proxies for processes and therefore help hindcast changes in ecosystem drivers. Relevant state metrics can be identified and interpreted using a resilience perspective to understand reef dynamics and assist with the interpretation of future reef trajectories based on mechanistic ecological relationships. The development of protocols on how state metrics can be interpreted to identify potential drivers and to estimate reef recovery based on ecological knowledge has recently been demonstrated by Flower et al. [53]. For instance, a decrease in juvenile coral density may imply a reduction of substrate availability, larval supply, or the occurrence of disturbance. However, if turf canopy height is also found to increase, a diagnosis can be refined suggesting that the substrate is becoming less hospitable for recruitment, either because of reduced herbivory and/or greater rates of primary production (nutrients). With appropriate tools and analyses, valuable information collected from historical monitoring programs can be used to apply RBM to coral reefs.

In all types of surveys, there exists a trade-off between the speed and ease of data collection, and the level of taxonomic resolution. Our results show that resilience assessments have deviated from documenting detailed changes in the biological community to focusing on a few metrics representing key reef processes [37]. Examples include documenting coral functional groups instead of species [58], and omitting groups of organisms that have less well understood ecosystem roles such as sponges, ascidians and tunicates [37]. Functional approaches were developed for coral reef fishes and algae, where species level is either too difficult to acquire, or so variable that it imparts little information [39, 59]. Resilience assessments tend to take a functional approach to the benthos, suggesting an implicit assumption that species-specific measurements are less relevant, such as focusing on branching coral cover [58] and in some cases, only using coral cover in total [37, 50].

Many high taxonomic level metrics currently assessed by monitoring programs contain valuable information for reef management and should not be left out [53]. For example, if management is focused on conserving biodiversity, then species level taxonomic resolution is a necessity. While higher-level taxonomic data is more time consuming to collect, the data can be combined into functional groups if desired. Detailed benthic documentation is important even when simplified to major lifeforms, especially when results are linked to ecosystem functioning such as the critical role of plating corals in driving rapid reef recovery in the Pacific [60–62] and the importance of branching *Acropora* as a nursery habitat for juvenile fishes [63]. The attempt to make resilience assessments logistically more feasible with lower resolution data may limit the insights that can be drawn with respect to reef functioning. To provide a comprehensive understanding for current and future RBM, it is critical to work out an optimal strategy to find a balance between monitoring state vs. process metrics, and the level of taxonomic resolution [57]. A potential approach would be to adopt as detailed taxonomic resolution as is possible for some of the most important ecological processes driving resilience and compromise the taxonomic resolution on others.

### Avenues for future research

Two of the continuing challenges for reef monitoring, whether with a taxonomic or more resilience-based focus, are incentivising data collection and linking the results to management actions. Greater synthesis and interpretation of monitoring data could help resolve both problems by adding interpretative value to the products. Currently, many monitoring programmes archive data, and while overall trends are plotted, there is often limited interpretation of patterns. Are the trends of concern or merely natural fluctuations? Can the trends be used to diagnose a problem and suggest a management intervention? Greater efforts to provide a diagnostic and interpretative interface, including the use of decision trees, can enhance the usefulness of data [53]. Here, the incorporation of process-orientated metrics along with changes in state can help provide diagnostic power.

Another opportunity to enhance the usefulness of monitoring data is in understanding and predicting reef dynamics. For example, statistical models such as Bayesian Belief Networks (BBN) [64, 65] can draw on individual case datasets to build predictive models. The more diverse the input data, in terms of environments and ecosystem states sampled, the stronger the predictions become. In an ideal scenario for coral reef monitoring data, it would be possible to imagine a central coral reef BBN being updated by multiple sets, with data providing new combinations of reef state and process and the ensuing trajectories. Individual practitioners could add their data and benefit from the functionality of the full reef model. A desirable outcome of this approach is that monitoring data are used to construct a dynamic model of the ecosystem that allows managers and scientists to run ‘what if’ scenarios, such as the likely consequences of reducing nutrient concentrations influencing reefs of a particular state and in a particular environment [66].

While utilising a global knowledge-base should improve predictions of reef state, it clearly raises concerns over heterogeneity in reef functioning. For example, processes influencing resilience, including the bottom-up and top-down drivers of macroalgal populations and the sensitivity of corals to algal interactions, can vary enormously between major biogeographic regions [67, 68]. Therefore, there remains a need for studies to identify the proximate drivers of reef dynamics across a diversity of environments [69]. Some of this can be achieved through globally-replicated experiments [68, 70] and regional analyses of bivariate relationships [71, 72], but it is critical that key processes are disaggregated and not lost as confounding effects in broad-scale studies [73].

This paper is written for both scientists and managers to identify promising new directions for RBM. There exists a wealth of monitoring data from different parts of the world that is highly fragmented and has rarely been analysed comprehensively [28]. While not specifically designed to measure processes, many monitoring variables provide a proxy of process. By using statistical approaches that consolidate monitoring data and hindcast trends in reef health [74, 75], comparisons can be drawn between the observed trajectory of reefs and those predicted from novel metrics-orientated approaches.

## Supporting information

S1 Checklist PRIMSA Checklist for current study. (PDF)

S1 Table The rationale behind the classification of studies into monitoring programs or resilience assessments. (PDF)

S2 Table Original metrics used in studies and their respective classification in the three phases of analysis. The standard metric, higher-level metric and metric categories were used in Figs 1–3 respectively.
(PDF)

S3 Table Results of two-way permutational MANOVA and PERMDISP test of homogeneity of dispersions between monitoring and resilience studies. (PDF)

S4 Table Results of SIMPER analysis for studies within the monitoring group (A), resilience group (B) and a comparison between the two groups (C).
(PDF)
