# Supplementary material for: The influence of resilience-based management on coral reef monitoring: A systematic review
Source: PLoS One. 2017 Feb 10;12(2):e0172064. doi: 10.1371/journal.pone.0172064 (PMC5302802; doi:10.1371/journal.pone.0172064)
Supplement: S1 Table — (PDF) [file pone.0172064.s002.pdf]

S1 Table. The rationale behind the classification of studies into monitoring programs and resilience assessments.

Study/Program	Stated goals	Rationale
1. The Atlantic and Gulf Rapid Reef Assessment (AGRRA)	Goal: Provide a database suitable for comparative evaluation of current reef condition with a focus on ancillary observation on reef distribution, community structure, geomorphology and identification of localised threats.<br><br>Approach: To characterise and compare reef conditions, distinguish between local and regional impacts and to recognise the effect of acute and chronic stressors on reef ecosystems through collecting data on key variables that reflect the structure and function of reef ecosystems.<br><br>Methods: Collect relatively simple quantitative indicators of reef condition, and abundance and trends at specific depth intervals.<br><br>Time period: 1997 – present<br><br>Reference: Lang et al. 2003	Despite being a well-known monitoring program, the AGRRA protocol differs from other monitoring programs with greater emphasis on the link between key variables and ecological processes to provide an indication of reef resilience. In addition, Brucker (2012) has stated AGRRA as one rapid ecological approach that can provide an indication of the reef resilience together with the IUCN resilience assessment protocol (Obura & Grimsditch 2009). Hence AGRRA is classified under resilience assessments for this analysis.
2. Australian Institute of Marine Science, Long-Term Monitoring Program (AIMS LTMP)	Objective: Monitor status of benthic coral reef communities to quantify percent cover changes in benthos over time<br><br>Time period: 1992 – present<br><br>Reference: Jonker et al. 2008	The AIMS LTMP protocol focuses on monitoring the status of benthic coral reef communities and cover changes, and hence is classified as a monitoring program.
3. Bruckner 2012	Objective: Determine the history of disturbance and resilience of reef sites using static measurements of resilience using a rapid assessment technique.<br><br>Approach: Focused on coral population dynamics and is a hybrid strategy between AGRRA and IUCN resilience protocols with a few modifications. Data collected in 2010.	This study focuses on variables that will help determine the resilience of a site, despite acknowledging its limits as a one-off study that relies on static metrics. This is classified as a resilience study based on the intention of the study.

	Methods: Characterise colony size structure, partial mortality, recruitment and whether juvenile corals represent growing recruits or remnant colonies.	
4. CARICOMP	Goal: A regional scientific program to study land-sea interaction in the Caribbean coastal zone and create a network of observers that can collaborate on region-wide events.<br><br>Approach: Record changes in coral reef ecosystems by the regular monitoring of physical and biological factors and to distinguish between natural and human impacts.<br><br>Time period: 1992 – present<br><br>Reference: Alcolado et al. 2001	CARICOMP is a long-term regional monitoring program in the Caribbean, with a focus on the regular recording of the changes in physical and biological parameters. Hence CARICOMP is classified as a monitoring program.
5. Cinner et al. 2013	Goal: To assess and compare the key ecological and social components of coastal social-ecological systems.<br><br>Approach: Used three categories including environmental exposure, ecological sensitivity and ecological recovery potential, to represent the ecological components of the coastal social-ecological system. Data collected in 2009, 2011-2012.<br><br>Methods: Developed metrics to represent key characteristics of ecological exposure, ecological sensitivity and recovery potential of reefs to bleaching. Recovery potential was represented by ten indicators of ecological sensitivity and recovery potential.	Although termed as a vulnerability study, this study developed metrics for ecological exposure, ecological sensitivity and recovery potential of coral reef ecosystems which are compatible with the resistance and recovery concepts in resilience. In addition, the study used the resilience metrics weighting proposed by McClanahan et al. (2012), hence is classified as a resilience study using the relevant ecological metrics.
6. CRAMP	Goal: Established in 1998 to monitor the long-term changes of reef benthic communities in Hawaii, and to evaluate the reef condition.	CRAMP focuses on repeated measurements of organisms and environmental parameters to evaluate the condition of reef communities in Hawaii, and hence is classified as a monitoring program.

	Methods: Monitoring of the abundance and cover of organisms, in addition to environmental parameters at permanent sites over time.<br><br>Time period: 1998 – present<br><br>Reference: Jokiel et al. 2001	
7. CREMP	Goal: To monitor trends of reefs in the Florida Keys National Marine Sanctuary (FKNMS).<br><br>Methods: Monitoring assessments conducted annually at permanent sites on changes in benthic cover and diversity of corals and other reef organisms<br><br>Time period: 1996 – present<br><br>Reference: Ruzicka et al. 2009	CREMP focuses on the collection of state metrics such as changes in benthic cover, trends and diversity, and hence is classified as a monitoring program.
8. Graham et al. 2015	Goal: To use site-level factors to predict whether sites would experience a regime-shift or recovery.<br><br>Methods: Site factors were chosen based on evidence from literature that targets key processes or key roles in structuring coral reef ecosystems globally. Data collected in 1994, 1998, 2005, 2008 and 2011.	In this study, 11 factors were chosen based on existing literature to represent key processes in coral reef ecosystems that will allow the prediction of a regime-shift. As resilience can be defined as the probability of a system shifting into a different regime, this is classified as a resilience study.
9. Jouffray et al. 2015	Goal: To detect and define potential multiple ecosystem regimes using a comprehensive coral reef dataset in Hawaii.<br><br>Methods: Tested and identified key human and environmental variables that were associated with different coral reef regimes. Data collected in 2010.	A number of predictor variables were identified to define multiple ecosystem regimes in a Hawaii coral reef ecosystem in this study. As resilience can be defined as the probability of a system to shifting into a different regime, this is classified as a resilience study.
10. Maynard et al. 2010	Goal: To develop a framework towards the operationalisation of coral reef resilience.	This paper introduces a framework that outlines metrics for assessing resilience, and hence is classified as a resilience study.

	Methods: Identified 19 indicators of coral reef resilience with weighting based on evidence and management effectiveness. The framework provides a method to generate scores for individual sites based on the evaluation of the identified indicators. Data collected in 2007, 2008.	
11. McClanahan et al. 2012	Goal: To develop selection criteria for prioritising coral reef resilience indicators and to identify reefs with the greatest resilience to climate disturbance.<br><br>Methods: Analysed a suite of resilience indicators and Identified priority resilience indicators based on scientific evidence and expert opinion.	The goal of the study is to prioritise key indicators for quantifying resilience based on literature evidence and expert opinion, and hence is classified as a resilience study.
12. Mumby et al. 2014	Goal: To quantify the ecological resilience of the Belize barrier reef under different scenarios of climate change.<br><br>Methods: Integrated biophysical data and modelled complex interactions among coral reef processes and different types of disturbance. The model was used to measure/calculate resilience, which is defined as the probability that a reef remained in the coral-dominated regime.	This paper uses biophysical data to model ecological processes and disturbances to derive measurements of resilience for reefs in Belize, and hence is classified as a resilience study.
13. NOAA Center for Coastal Monitoring and Assessment, Biogeography Branch	Goal: To characterise and monitor the distribution, abundance and size of marine coral reef organisms and develop data collection and management protocols.<br><br>Methods: Long-term measurements of coral reef organisms and relate it with habitat data to establish a knowledge base.<br><br>Time period: 1990 – present<br><br>Reference: Pittman et al. 2010	NOAA's Biogeography Branch focuses on monitoring state metrics such as disturbance, abundance and size of marine fauna found in reefs, and hence is classified as a monitoring program.
14. Obura & Grimsditch 2009 (IUCN)	Goal: To develop protocols to understand resistance and resilience indicators for management applications and to determine how MPA management actions can affect a reef's resilience and resistance.	This report details a protocol developed by IUCN, identifying 61 metrics that can be used to assess coral reef resilience, and hence is classified as a resilience study.

	Methods: A rapid assessment of resistance and resilience indicators at individual site levels and to assess past management actions in the maintenance of reef resilience.	
15. Reef Check	Goal: To survey and assess the health of coral reefs globally with a standardised method to allow comparisons of survey results.<br><br>Methods: Focus on the abundance of specific reef organisms that reflect the condition of reef ecosystem and are easily recognizable to the public.<br><br>Time period: 1996 – present<br><br>Reference: Hodgson et al. 2006	Reef check is a global monitoring program designed to allow the public to collect information and document coral reef ecosystem change. It is focused on the abundance and trends of key reef organisms, and hence is classified as a monitoring program.
16. Rowlands et al. 2012	Goal: To develop a method to use remote sensing data to estimate coral reef resilience and to measure the differences in resilience of different reef sites in the Red Sea.<br><br>Methods: Indices are used to utilise remote-sensing data to quantify resilience, and are divided between factors that stress, contribute to resistance or promote coral growth and recovery.	This goal of this study is to develop a method to quantify resilience using remote-sensing data, and hence is classified as a resilience study.
17. Weeks & Jupiter 2013	Goal: To identify critical areas for reef resilience that would be suitable for adaptive co-management by establishing marine protected areas. Data collected in 2005.<br><br>Methods: Identified site-level indicators for reef resilience based on literature and conducted surveys to collect data that were analysed to produce a single resilience score for individual sites.	Despite being termed as an adaptive co-management study on marine protected areas, one component of this study identified critical areas for reef resilience by using site-level indicators of coral reef resilience and hence is classified as a resilience study.
18. West & Salm 2003	Goal: Identify factors of resistance and resilience to identify reef areas that most likely to be resilient to climate change.	This paper identifies metrics that are related to the resistance and recovery of corals, and hence is classified as a resilience study.

	Methods: Proposed indicators that are related to factors that reduce stress and environmental factors that enhance coral resistance and recovery.	

References:

1. Lang JC. Status of coral reefs in the western Atlantic: Results of initial surveys, Atlantic and Gulf Rapid Reef Assessment (AGRRA) program. Atoll Res Bull. 2003;496.
2. Jonker M, Johns K, Osborne K. Surveys of Benthic Reef Communities Using Digital Photography and counts of Juvenile Corals. Townsville: Australian Institute of Marine Science, 2008.
3. Bruckner AW. Static measurements of the resilience of Caribbean coral populations. Rev Biol Trop. 2012;60:39-57.
4. Obura D, Grimsditch G. Resilience assessment of coral reefs: assessment protocol for coral reefs, focusing on coral bleaching and thermal stress. Gland, Switzerland: IUCN; 2009.
5. Alcolado PM, Alleng G, Bonair K, Bone D, Buchan K, Bush PG, et al. The Caribbean Coastal Marine Productivity Program (CARICOMP). Bull Mar Sci. 2001;69(2):819-29.
6. Cinner JE, Huchery C, Darling ES, Humphries AT, Graham NAJ, Hicks CC, et al. Evaluating Social and Ecological Vulnerability of Coral Reef Fisheries to Climate Change. PLoS ONE. 2013;8(9):e74321.
7. Jokiel PL, Brown EK, Friedlander AM, Rodgers SK, Smith WR. Hawaii Coral Reef Initiative Coral Reef Assessment and Monitoring Program (CRAMP) Final Report 1999-2000. Hawaii Institute of Marine Biology, Oceanic Institute and NOAA, 2001.
8. Ruzicka R, Colella M, Semon K, Brinkhuis V, Morrison J, Kidney J, et al. CREMP 2009 Final Report. Saint Petersburg, FL: Fish & Wildlife Research Institute/Florida Fish & Wildlife Conservation Commission, 2009.
9. Graham NAJ, Jennings S, MacNeil MA, Mouillot D, Wilson SK. Predicting climate-driven regime shifts versus rebound potential in coral reefs. Nature. 2015;518(7537):94-7.
10. Jouffray JB, Nystrom M, Norstrom AV, Williams ID, Wedding LM, Kittinger JN, et al. Identifying multiple coral reef regimes and their drivers across the Hawaiian archipelago. Philosophical Transactions of the Royal Society B-Biological Sciences. 2015;370(1659).
11. Maynard JA, Marshall PA, Johnson JE, Harman S. Building resilience into practical conservation: identifying local management responses to global climate change in the southern Great Barrier Reef. Coral Reefs. 2010;29(2):381-91.
12. McClanahan TR, Campbell SJ, Darling ES, Eakin CM, Heron SF, Jupiter SD, et al. Prioritizing key resilience indicators to support coral reef management in a changing climate. PLoS ONE. 2012;7(8):e42884.
13. Mumby PJ, Wolff NH, Bozec YM, Chollett I, Halloran P. Operationalizing the resilience of coral reefs in an era of climate change. Conservation Letters. 2014;4(7):1-12.
14. Pitmann SJ, Hile SD, Jeffrey CFG, Clark R, Woody K, Herlach BD, et al. Coral reef ecosystems of Reserva Natural La Parguera (Puerto Rico): Spatial and temporal patterns in fish and benthic communities (2001-2007). NOAA Technical Memorandum NOS NCCOS 107. Silver Spring, MD: 2010.
15. Hodgson G, Hill J, Kiene W, Maun L, Mihaly J, Liebeler J, et al. Reef check instruction manual: A guide to reef check coral reef monitoring. Pacific Palisades, California, USA: Reef Check Foundation; 2006. 87 p.
16. Rowlands G, Purkis S, Riegl B, Metsamaa L, Bruckner A, Renaud P. Satellite imaging coral reef resilience at regional scale. A case-study from Saudi Arabia. Mar Pollut Bull. 2012;64(6):1222-37.

17. Weeks R, Jupiter SD. Adaptive Comanagement of a Marine Protected Area Network in Fiji. Conserv Biol. 2013;27(6):1234-44.
18. West JM, Salm RV. Resistance and resilience to coral bleaching: implications for coral reef conservation and management. Conserv Biol. 2003;17(4):956-67.
