# Supplementary material for: The influence of resilience-based management on coral reef monitoring: A systematic review
Source: PLoS One. 2017 Feb 10;12(2):e0172064. doi: 10.1371/journal.pone.0172064 (PMC5302802; doi:10.1371/journal.pone.0172064)
Supplement: S2 Table — The standard metric, higher-level metric and metric categories were used in Figs 1–3 respectively. (PDF) [file pone.0172064.s003.pdf]

S2 Table. Original metrics used in studies and their respective classification in the three phases of analysis. The standard metric, higher-level metric and metric categories were used in Figs 1-3 respectively.

#	Original metric	Standard metric	Higher-level metric	Metric Categories	Additional information on metric	Reference
1	Latitude and longitude	Latitude/Longitude	Location & Geomorphology	State		AGRRA
2	Reef type	Reef type	Location & Geomorphology	State	Zones of maximum reef development are highly recommended: shallow reef crests (<5m), intermediate depth (5-15m) linear or patch reefs. Other examples are atolls, fringing and barrier reefs, etc.	AGRRA
3	Site exposure (windward or leeward)	Site exposure	Location & Geomorphology	State/Proxy	Location with respect to the normal wind patterns (windward, leeward, seasonally variable) and open ocean	AGRRA
4	Reef zone	Reef zone	Location & Geomorphology	State	Geomorphic zone of a reef on an insular or continental shelf: shore, bay, lagoon, intertidal crest, channel, forereef, etc.	AGRRA
5	Reef subtype	Reef subtype	Location & Geomorphology	State	Thicket, reticulate, coral field, rubble field, sand/mud (not a real reef)	AGRRA
6	Angle relative to horizontal	Slope	Location & Geomorphology	State	Flat, moderate slope, steep slope, wall, etc.	AGRRA
7	Vertical relief	Relief	Location & Geomorphology	State		AGRRA
8	Management	Management	Management Status	State/Proxy		AGRRA
9	Depth	Depth	Location & Geomorphology	State		AGRRA
10	Temperature	Temperature	Physical characteristics	State/Proxy		AGRRA
11	Live coral by species (or genus)	Coral cover (genus)	Coral cover	State	Primary reef constructors, universal reef condition indicator	AGRRA

12	Species diversity	Coral diversity	Coral community	State		AGRRA
13	Bleached coral cover	Bleached coral cover	Disturbance (Physiological)	State/Proxy	Indicates altered reef conditions (often thermal stress when large scale)	AGRRA
14	Pale live coral cover	Paling coral cover	Disturbance (Physiological)	State/Proxy	Indicates beginning of bleaching, or beginning of recovery after having bleached	AGRRA
15	Newly dead coral cover	Dead coral cover	Mortality	State/Proxy	Indicates ongoing disease, bleaching, predation, competition, or other perturbations	AGRRA
16	CCA	CCA	Algal cover	State/Proxy	Cement broken corals together to make reef framework, may indicate good conditions for coral recruits to settle	AGRRA
17	Newly dead CCA	CCA Dead	Algal cover	State		AGRRA
18	Calcified worm tubes	Substrate	Substrate	State		AGRRA
19	Microbial and/or microalgal biofilm	Biofilm	Algal cover	State/Proxy	When corals die, skeletons quickly covered with biofilms of bacteria/microalgae, often mistaken as bare space. Coral larvae can settle here.	AGRRA
20	Cyanobacteria cover	Cyanobacteria	Algal cover	State	Photosynthetic organisms that compete with corals for space on reefs, may also shelter pathogens that can kill corals	AGRRA
21	Sparse turf algae	Turf	Algal cover	State	Eaten by many herbivorous fishes and *Diadema*	AGRRA
22	Turf algal mat (TAM)	Turf algal mat	Herbivory	State/Proxy	Indicates few herbivores, this prevents coral larvae from settling and overgrows corals and CCA. May also shelter pathogens that kills corals	AGRRA
23	Turf algal sediment mat	Turf algal sediment mat	Algal cover	State/Proxy	Not eaten by herbivores, inhibit settlement of coral larvae, overgrow corals and CCA. May shelter pathogens that kill corals.	AGRRA
24	Fleshy/calcareous peyssonnelid	Peyssonnelid	Algal cover	State	Inhibits settlement, unlike other CCA. Some species overgrow corals and CCA.	AGRRA
25	Fleshy macroalgae	Macroalgae	Algal cover	State/Proxy	Eaten by some herbivorous fishes, some overgrow corals and CCA, inhibit coral larvae from settling and shelter pathogens that kill corals	AGRRA
26	Calcareous macroalgae	Coralline algae	Algal cover	State	Eaten by some herbivorous fishes, some overgrow corals and CCA, inhibit coral larvae from settling and shelter pathogens that kill corals	AGRRA

27	Macroalgal height	Algal height	Herbivory	State/Proxy	MA volume = cover x height, proxy for biomass, is inversely related to herbivory	AGRRA
28	Newly dead macroalgae	Macroalgae Dead	Algal cover	State		AGRRA
29	Aggressive invertebrates - *Cliona delitrix*, zoozanthellate *Cliona*	Harmful organisms	Organisms (Harmful)	State/Proxy	Kill and expand over corals and CCA as they grow	AGRRA
30	Aggressive invertebrates - *Chondrilla caribensis*	Harmful organisms	Organisms (Harmful)	State/Proxy	Kill and expand over corals and CCA as they grow	AGRRA
31	Aggressive invertebrates - *Millepora alcicornis*	Harmful organisms	Organisms (Harmful)	State/Proxy	Kill and expand over corals and CCA as they grow	AGRRA
32	Aggressive invertebrates - *Briarium asbestinum, Erythropodium caribaeorum*	Harmful organisms	Organisms (Harmful)	State/Proxy	Kill and expand over corals and CCA as they grow	AGRRA
33	Aggressive invertebrates - *Palythoa caribaeorum, Tridudemnum solidum*	Harmful organisms	Organisms (Harmful)	State/Proxy	Kill and expand over corals and CCA as they grow	AGRRA
34	Sessile invertebrates - Epibenthic sponge	Other Invertebrates (Sessile)	Other invertebrates	State	Usually don't kill and expansively overgrow corals and CCA	AGRRA
35	Sessile invertebrates - Gorgonian	Other Invertebrates (Sessile)	Other invertebrates	State	Usually don't kill and expansively overgrow corals and CCA	AGRRA
36	Non-living - Crevice	Substrate	Substrate	State	Coral larvae can settle here	AGRRA
37	Non-living - Sand, mud, sand-mud mix	Substrate	Substrate	State		AGRRA
38	Substratum - live coral	Substrate	Substrate	State/Proxy	Helps characterise past history of the site	AGRRA
39	Substratum - dead coral	Substrate	Substrate	State/Proxy	Helps characterise past history of the site	AGRRA
40	Substratum - coral rubble, dead coral boulder	Substrate	Substrate	State/Proxy	Helps characterise past history of the site	AGRRA

41	Substratum - carbonate pavement	Substrate	Substrate	State/Proxy	Helps characterise past history of the site	AGRRA
42	Small coral recruits (<2cm), large coral recruits (2-4 cm)	Juvenile corals	Recruitment	State/Proxy	Recruits are counted to learn if reefs might be able to recover when disasters kill adult corals	AGRRA
43	*Diadema antillarum* (juvenile and adult)	Urchin	Herbivory	State/Proxy	Formerly a key herbivore on many W. Atlantic reefs	AGRRA
44	Spiny lobsters	Motile invertebrates	Other invertebrates	State	Overfished across much of the W. Atlantic	AGRRA
45	Live queen conch	Motile invertebrates	Other invertebrates	State/Proxy	Usually seen only on reefs that have not been overfished	AGRRA
46	Lionfish	Lionfish	Disturbance (Biological)	State/Proxy	Invasive and predatory, preying upon and competing with many native species	AGRRA
47	Trash	Garbage	Disturbance (Anthropogenic)	State/Proxy	Discards reflect human behaviour and activity e.g. boating, fishing, drinking	AGRRA
48	Coral disease	Disease	Disturbance (Physiological)	State/Proxy		AGRRA
49	Coral size	Coral size	Coral community	State/Proxy	Small corals likely to be completely killed by perturbations. A reef without large corals may be recovering from a serious perturbation, one without recruits has no future	AGRRA
50	Fish abundance (Selected functional group)	Fish abundance (key)	Fish abundance	State		AGRRA
51	Fish abundance (Selected species)	Fish abundance (key)	Fish abundance	State		AGRRA
52	Fish size frequency distribution (selected species)	Fish size (key)	Fish size	State		AGRRA
53	Fish diversity	Fish diversity	Fish community	State		AGRRA

54	Herbivorous fish biomass (species)	Herbivore biomass	Herbivory	State/Proxy		AGRRA
55	Fish biomass (estimated)	Fish biomass	Fish biomass	State/Proxy	Total fish biomass gives important information on trophic structure and overall reproductive output of fish	AGRRA
56	Commerical fish biomass (estimated)	Fish biomass	Fish biomass	State/Proxy	Total commercial fish biomass gives indication of stock status, fishing pressure, habitat conditions and recruitment success	AGRRA
57	Crustose coralline algae cover	CCA	Algal cover	State		AIMS LTMP
58	Macroalgae cover	Macroalgae	Algal cover	State		AIMS LTMP
59	Turf algae cover	Turf	Algal cover	State		AIMS LTMP
60	*Halimeda*	Coralline algae	Algal cover	State		AIMS LTMP
61	Bleached coral cover	Bleached coral cover	Disturbance (Physiological)	State/Proxy		AIMS LTMP
62	Giant clam abundance	Motile invertebrates	Other invertebrates	State		AIMS LTMP
63	Coral cover (Genus)	Coral cover (genus)	Coral cover	State		AIMS LTMP
64	Dead coral cover	Dead coral cover	Coral cover	State		AIMS LTMP
65	Hard coral cover	Coral cover	Coral cover	State		AIMS LTMP
66	COTS abundance	COTS	Disturbance (Biological)	State/Proxy		AIMS LTMP
67	COTS size class distribution	COTS	Disturbance (Biological)	State/Proxy		AIMS LTMP
68	Presence of feeding scars	Feeding scars	Disturbance (Biological)	State/Proxy		AIMS LTMP
69	Coral disease	Disease	Disturbance	State/Proxy		AIMS LTMP

			(Physiological)			
70	Total fish abundance	Total fish abundance	Fish abundance	State		AIMS LTMP
71	Fish diversity	Fish diversity	Fish community	State		AIMS LTMP
72	Fish size frequency distribution (Selected species)	Fish size (key)	Fish size	State		AIMS LTMP
73	Soft coral cover	Other Invertebrates (Sessile)	Other invertebrates	State		AIMS LTMP
74	Sponge cover	Other Invertebrates (Sessile)	Other invertebrates	State		AIMS LTMP
75	Visibility	Turbidity	Physical characteristics	State/Proxy	Water visibility estimates recorded.	AIMS LTMP
76	Tide	Tide	Physical characteristics	State	Tide recorded as low, high, falling or rising, determined from a tide table.	AIMS LTMP
77	Cloud cover	Cloud cover	Physical characteristics	State	Quantified in terms of eighths of the sky area covered by cloud. Unit = okta. One okta means 1/8 of the sky.	AIMS LTMP
78	Wind strength	Wind	Physical characteristics	State/Proxy	5 categories from 0-25 knots.	AIMS LTMP
79	Disease	Disease	Disturbance (Physiological)	State/Proxy	Presence/absence	AIMS LTMP
80	Cyclones	Storm	Disturbance (Physical)	State/Proxy	Presence/absence	AIMS LTMP
81	Abiotic cover	Substrate	Substrate	State		AIMS LTMP
82	Juvenile corals	Juvenile corals	Recruitment	State/Proxy	Up to 5cm are surveyed. Juvenile corals are the net result of processes of larval supply, settlement and post-settlement survivorship.	AIMS LTMP
83	Total Carbon	Carbon	Disturbance	State/Proxy		AIMS LTMP

			(Anthropogenic)			
84	Total Nitrogen	Nitrogen	Disturbance (Anthropogenic)	State/Proxy		AIMS LTMP
85	Temperature	Temperature	Physical characteristics	State/Proxy		AIMS LTMP
86	Suspended solids	Turbidity	Physical characteristics	State/Proxy		AIMS LTMP
87	Salinity	Salinity	Physical characteristics	State		AIMS LTMP
88	Chlorophyll-a	Chlorophyll	Disturbance (Anthropogenic)	State/Proxy		AIMS LTMP
89	Fleshy macroalgae	Macroalgae	Algal cover	State		Bruckner 2012
90	Erect coralline algae	Coralline algae	Algal cover	State		Bruckner 2012
91	Crustose coralline algae	CCA	Algal cover	State		Bruckner 2012
92	Turf algae	Turf	Algal cover	State		Bruckner 2012
93	Cyanobacteria	Cyanobacteria	Algal cover	State		Bruckner 2012
94	Coral cover (species)	Coral cover (species)	Coral cover	State		Bruckner 2012
95	Coral diversity	Coral diversity	Coral community	State		Bruckner 2012
96	Coral size class distribution	Coral size	Coral community	State/Proxy		Bruckner 2012
97	Tunicate	Other Invertebrates (Sessile)	Other invertebrates	State		Bruckner 2012
98	Gorgonian	Other Invertebrates (Sessile)	Other invertebrates	State		Bruckner 2012

99	Anemones	Other Invertebrates (Sessile)	Other invertebrates	State		Bruckner 2012
100	Bioeroding sponges	Other Invertebrates (Sessile)	Other invertebrates	State		Bruckner 2012
101	Hydrozoan coral (*Millepora*)	Other Invertebrates (Sessile)	Other invertebrates	State		Bruckner 2012
102	Pavement	Substrate	Substrate	State		Bruckner 2012
103	Rubble	Substrate	Substrate	State		Bruckner 2012
104	Sand/Silt	Substrate	Substrate	State		Bruckner 2012
105	Recently dead coral cover (Coral condition)	Dead coral cover	Mortality	State/Proxy	Visual estimates of tissue loss	Bruckner 2012
106	Transitional dead coral cover (Coral condition)	Dead coral cover	Coral cover	State	Visual estimates of tissue loss	Bruckner 2012
107	Old dead coral cover (Coral condition)	Dead coral cover	Coral cover	State	Visual estimates of tissue loss	Bruckner 2012
108	Cause of mortality	Cause of mortality	Mortality	State/Proxy	Assessment of type of disease, extent of bleaching, predation, competition, overgrowth or other cause of mortality	Bruckner 2012
109	Juvenile corals	Juvenile corals	Recruitment	State/Proxy	Sampling for corals smaller than 4cm, divided into 2 classes	Bruckner 2012
110	Settlement location	Settlement location	Recruitment	State/Proxy	Found M. *annularis* skeletons promoted settlement.	Bruckner 2012
111	Longitude/latitude	Latitude/Longitude	Location & Geomorphology	State		Bruckner 2012
112	Key nuisance invertebrates (Sponge, gorgonian, bioeroding sponge, tunicate)	Harmful organisms	Organisms (Harmful)	State/Proxy	Nuisance species: tunicate (*Tredidemnum*), encrusting gorgonian (*Erythropodium, Briareum*), colonial anemone (*Palythoa*), encrusting or bioeroding sponge (*Cliona*) and hydrozoan coral (*Millepora*)	Bruckner 2012
113	Disease	Disease	Disturbance (Physiological)	State/Proxy		Bruckner 2012

114	Bleached coral cover	Bleached coral cover	Disturbance (Physiological)	State/Proxy		CARICOMP
115	Algal biomass (Select species)	Algal biomass	Algal biomass	State/Proxy		CARICOMP
116	Calcareous algae	Coralline algae	Algal cover	State		CARICOMP
117	Encrusting calcareous algae	CCA	Algal cover	State		CARICOMP
118	Fleshy Algae	Macroalgae	Algal cover	State		CARICOMP
119	Turf algae	Turf	Algal cover	State		CARICOMP
120	Anemones	Other Invertebrates (Sessile)	Other invertebrates	State		CARICOMP
121	Ascidians	Other Invertebrates (Sessile)	Other invertebrates	State		CARICOMP
122	Branching corals	Coral cover (growth form)	Coral community	State/Proxy		CARICOMP
123	Massive corals	Coral cover (growth form)	Coral community	State/Proxy		CARICOMP
124	Encrusting corals	Coral cover (growth form)	Coral community	State/Proxy		CARICOMP
125	Foliaceous corals	Coral cover (growth form)	Coral community	State/Proxy		CARICOMP
126	Milleporines	Other Invertebrates (Sessile)	Other invertebrates	State		CARICOMP
127	Recently dead coral	Dead coral cover	Mortality	State/Proxy		CARICOMP
128	Hard coral	Coral cover	Coral cover	State		CARICOMP
129	Coral disease	Disease	Disturbance	State/Proxy		CARICOMP

			(Physiological)			
130	Corallimorpharians	Other Invertebrates (Sessile)	Other invertebrates	State		CARICOMP
131	Fish size frequency distribution by species	Fish size (species)	Fish size	State		CARICOMP
132	Fish abundance (Selected species)	Fish abundance (key)	Fish abundance	State		CARICOMP
133	Fish diversity (Species)	Fish diversity	Fish community	State		CARICOMP
134	Gorgonians (Growth form)	Other Invertebrates (Sessile)	Other invertebrates	State		CARICOMP
135	Encrusting gorgonians	Other Invertebrates (Sessile)	Other invertebrates	State		CARICOMP
136	Bare boulders	Substrate	Substrate	State		CARICOMP
137	Bare rock	Substrate	Substrate	State		CARICOMP
138	Bare rubble	Substrate	Substrate	State		CARICOMP
139	Bare sediment	Substrate	Substrate	State		CARICOMP
140	Holes, gaps, overhangs	Substrate	Substrate	State		CARICOMP
141	Depth	Depth	Location & Geomorphology	State		CARICOMP
142	Latitude and longitude	Latitude/Longitude	Location & Geomorphology	State		CARICOMP
143	Rugosity	Rugosity	Location & Geomorphology	State/Proxy		CARICOMP
144	Erect sponges	Other Invertebrates (Sessile)	Other invertebrates	State		CARICOMP

145	Encrusting sponges	Other Invertebrates (Sessile)	Other invertebrates	State		CARICOMP
146	Sea surface temperature	Temperature	Physical characteristics	State/Proxy		CARICOMP
147	Urchin abundance (Species)	Urchin	Other invertebrates	State/Proxy		CARICOMP
148	Urchin abundance (juvenile)	Urchin	Other invertebrates	State/Proxy		CARICOMP
149	Urchin abundance (adult)	Urchin	Other invertebrates	State/Proxy		CARICOMP
150	Sea urchin density	Urchin	Herbivory	State/Proxy	As a key herbivore, potential of organism to reach high densities will change algal cover.	CARICOMP
151	Sea urchin size class distribution	Urchin	Other invertebrates	State/Proxy		CARICOMP
152	Salinity	Salinity	Physical characteristics	State		CARICOMP
153	Turbidity	Turbidity	Physical characteristics	State/Proxy		CARICOMP
154	Zoanthids	Other Invertebrates (Sessile)	Other invertebrates	State		Cinner et al. 2013
155	Ecological exposure to coral bleaching	Thermal regime	Disturbance (Physiological)	Process	Multivariate model of how variables combine to create environmental conditions that make a site susceptible to coral bleaching impacts including temperature, lights, currents, tidal variation, chlorophyll and water quality	Cinner et al. 2013
156	Temperature	Temperature	Physical characteristics	State/Proxy	Variable included in the multivariate model that produces ecological exposure resilience index	Cinner et al. 2013
157	Light	Light	Physical characteristics	State/Proxy	Variable included in the multivariate model that produces ecological exposure resilience index	Cinner et al. 2013
158	Currents	Current	Physical characteristics	Proxy	Variable included in the multivariate model that produces ecological exposure resilience index	Cinner et al. 2013

159	Tidal variation	Tide	Physical characteristics	State/Proxy	Variable included in the multivariate model that produces ecological exposure resilience index	Cinner et al. 2013
160	Chlorophyll-a	Chlorophyll	Disturbance (Anthropogenic)	State/Proxy	Variable included in the multivariate model that produces ecological exposure resilience index	Cinner et al. 2013
161	Water quality	Water quality	Disturbance (Anthropogenic)	State/Proxy	Variable included in the multivariate model that produces ecological exposure resilience index	Cinner et al. 2013
162	Susceptibility of the coral community to bleaching	Bleaching	Disturbance (Physiological)	Process		Cinner et al. 2013
163	Susceptibility of the fish community to population declines associated with habitat loss from bleaching	Bleaching	Disturbance (Physiological)	Process		Cinner et al. 2013
164	Hard coral cover	Coral cover	Coral cover	State	Linked to increase resilience and recovery but most studies show no correlation	Cinner et al. 2013
165	Ratio of coral to macroalgal cover	Coral-algal balance	Competition	State/Proxy		Cinner et al. 2013
166	Macroalgae cover	Macroalgae	Algal cover	State/Proxy	Limit coral recovery via competition for benthic substrate, allelopathy and trapping sediment that smothers coral recruits	Cinner et al. 2013
167	Coral size distribution	Coral size	Coral community	State/Proxy	Evidence that evenness across size classes increases recovery. Lack of large adult colonies may limit spawning stock.	Cinner et al. 2013
168	Coral richness	Coral diversity	Coral community	State	Expected to promote recovery, but limited evidence.	Cinner et al. 2013
169	Fish biomass (estimated)	Fish biomass	Fish biomass	State/Proxy	Indicates status of fish stock, it's potential growth and a proxy for ecological metabolism	Cinner et al. 2013
170	Fish species richness	Fish diversity	Fish community	State/Proxy	Often used as proxy for functional redundancy, expected to promote ecological recovery by avoiding undesired ecological states	Cinner et al. 2013
171	Fish size distribution	Fish size	Fish size	State/Proxy	Large individuals can increase fecundity to promote recovery of fish communities	Cinner et al. 2013
172	Substrate complexity	Rugosity	Location & Geomorphology	State/Proxy		Cinner et al. 2013

173	Herbivore diversity	Herbivore diversity	Fish community	State/Proxy		Cinner et al. 2013
174	Index of herbivore grazing relative to algal production	Grazing/Algal Production	Herbivory	Proxy		Cinner et al. 2013
175	Latitude and longitude	Latitude/Longitude	Location & Geomorphology	State		Cinner et al. 2013
176	Depth	Depth	Location & Geomorphology	State		Cinner et al. 2013
177	Calcifying to non-calcifying cover	Substrate availability	Substrate	State/Proxy	Calcifying organisms important for reef framework e.g. settlement, recruitment and cementation of reef structure, expect to increase recovery following disturbance	Cinner et al. 2013
178	CCA cover	CCA	Algal cover	State		CRAMP
179	Cyanobacteria cover	Cyanobacteria	Algal cover	State		CRAMP
180	Fleshy macroalgae	Macroalgae	Algal cover	State		CRAMP
181	Macroalgae	Macroalgae	Algal cover	State		CRAMP
182	Turf cover	Turf	Algal cover	State		CRAMP
183	Coral cover (Species)	Coral cover (species)	Coral cover	State		CRAMP
184	Coral mortality	Coral mortality	Mortality	Process		CRAMP
185	Coral richness	Coral diversity	Coral community	State		CRAMP
186	Total fish abundance	Total fish abundance	Fish abundance	State		CRAMP
187	Total fish biomass	Fish biomass	Fish biomass	State/Proxy		CRAMP
188	Fish size class distribution	Fish size	Fish size	State		CRAMP

189	Sediment size	Sediment size	Physical characteristics	State/Proxy	Used as an indicator of the environment and provide a characterisation of the benthos, suppling information about benthic structure	CRAMP
190	Coral recruitment	Juvenile corals	Recruitment	State/Proxy		CRAMP
191	Depth	Depth	Location & Geomorphology	State		CRAMP
192	Latitude and longitude	Latitude/Longitude	Location & Geomorphology	State		CRAMP
193	Rugosity	Rugosity	Location & Geomorphology	State/Proxy	More habitat for coral reef organisms, and proxy for fish abundance	CRAMP
194	Nutrients	Nutrients	Disturbance (Anthropogenic)	State/Proxy		CRAMP
195	Percent Carbon	Carbon	Disturbance (Anthropogenic)	State/Proxy		CRAMP
196	Percent Nitrogen	Nitrogen	Disturbance (Anthropogenic)	State/Proxy		CRAMP
197	Wave exposure	Wave exposure	Physical characteristics	Proxy		CRAMP
198	Conservation status	Management	Management Status	State/Proxy		CRAMP
199	Habitat	Habitat	Location & Geomorphology	State		CRAMP
200	Sand/Silt/Bare/Rock	Substrate	Substrate	State		CRAMP
201	Habitat type	Habitat	Location & Geomorphology	State	3 types: carbonate fringing, granitic rocky and patch reef. Habitats differ in coral recruitment success/likelihood of ecosystem collapse	Graham et al. 2015
202	Pre-disturbance branching coral cover	Coral cover (branching)	Coral cover	State/Proxy	Branching vulnerable to bleaching, and once dead erodes quickly. More branching vulnerable to coral loss and reduction in organisms	Graham et al. 2015
203	Juvenile coral density	Juvenile corals	Recruitment	State/Proxy	Proxy for successful recruitment, survival and growth of new corals, assumed to be key to coral recovery dynamics	Graham et al. 2015

204	Depth	Depth	Location & Geomorphology	State/Proxy	Threats on reefs are mostly in shallow water, light penetration also favour rapid growth of macroalgae	Graham et al. 2015
205	Herbivorous fish biomass	Herbivore biomass	Herbivory	State/Proxy	Good proxy for function as the area of reef grazed by fish to mediate competition for space among coral and algae	Graham et al. 2015
206	Herbivorous fish diversity	Herbivore diversity	Fish community	State/Proxy	Many types of algae have defences against herbivores, proxy for effective control of macroalgae	Graham et al. 2015
207	Marine reserve status	Management	Management Status	State/Proxy	No take reserves expect to reduce fishing and enhance ecosystem processes	Graham et al. 2015
208	Nutrient regime	Nutrients	Disturbance (Anthropogenic)	State/Proxy	High nutrient loads enhance growth of algae, resulting in algae outcompeting corals	Graham et al. 2015
209	Pre-disturbance structural complexity	Rugosity	Location & Geomorphology	State/Proxy	Provide refuge, and space for coral settlement and larvae. Prior to a disturbance, expect to maintain ecosystem processes through a disturbance	Graham et al. 2015
210	Sea urchin density	Urchin	Herbivory	State/Proxy	Sea urchins important herbivores to control algal growth, promote successful recruitment and recovery	Graham et al. 2015
211	Proximity to upwelling or exposure to strong currents	Upwelling	Physical characteristics	State/Proxy	Wave exposure influence distribution patterns, growth forms and colony sizes. Also enhance algal growth rates, and dislodge algae where wave exposure is strong	Graham et al. 2015
212	Coral cover (species)	Coral cover (species)	Coral cover	State		CREMP
213	Coral richness	Coral diversity	Coral community	State		CREMP
214	Octocoral cover (Gorgonian and soft coral)	Other Invertebrates (Sessile)	Other invertebrates	State		CREMP
215	Zoanthid cover	Other Invertebrates (Sessile)	Other invertebrates	State		CREMP
216	Sponge cover	Other Invertebrates (Sessile)	Other invertebrates	State		CREMP
217	Seagrass cover	Seagrass	Seagrass	State		CREMP

218	Macroalgae cover	Macroalgae	Algal cover	State		CREMP
219	Cyanobacteria cover	Cyanobacteria	Algal cover	State	Monitored in datasheets.	CREMP
220	Clionid sponge cover	Harmful organisms	Organisms (Harmful)	State/Proxy		CREMP
221	Bleached coral cover	Bleached coral cover	Disturbance (Physiological)	State/Proxy		CREMP
222	Disease	Disease	Disturbance (Physiological)	State/Proxy		CREMP
223	Habitat	Habitat	Location & Geomorphology	State	Four main habitat types: nearshore hardbottom, patch reefs, offshore shallow reefs and offshore deep reefs	CREMP
224	Temperature	Temperature	Physical characteristics	State/Proxy		CREMP
225	Diadema abundance/density	Urchin	Other invertebrates	State/Proxy		CREMP
226	Grazer biomass	Herbivore biomass	Herbivory	State/Proxy	Grazer crop on algal turf, prevent the establishment and growth of macroalgae	Jouffray et al. 2015
227	Scraper biomass	Herbivore biomass	Herbivory	State/Proxy	Scrapers also feed on turf but remove some component of the reef substratum, providing bare areas for coral recruitment	Jouffray et al. 2015
228	Browser biomass	Herbivore biomass	Herbivory	State/Proxy	Browsers consistently feed on macroalgae, may play a crucial role for reversing macroalgae-dominated states	Jouffray et al. 2015
229	Latitude	Latitude/Longitude	Location & Geomorphology	State		Jouffray et al. 2015
230	Depth	Depth	Location & Geomorphology	State		Jouffray et al. 2015
231	Distance from coast	Distance from coast	Disturbance (Anthropogenic)	Proxy		Jouffray et al. 2015
232	Distance from stream	Distance from stream	Disturbance (Anthropogenic)	Proxy		Jouffray et al. 2015

233	Effluent	Effluent	Disturbance (Anthropogenic)	Proxy		Jouffray et al. 2015
234	Human population density	Human population	Disturbance (Anthropogenic)	Proxy		Jouffray et al. 2015
235	Urban index	Urban disturbance	Disturbance (Anthropogenic)	Proxy	Representation of urban disturbance within a catchment	Jouffray et al. 2015
236	Point pollution index	Pollution	Disturbance (Anthropogenic)	State/Proxy	Representation of the density of sources of point pollution within a catchment (6 point sources of pollution)	Jouffray et al. 2015
237	Fragmentation index	Stream fragmentation	Disturbance (Anthropogenic)	Proxy	Representation of stream fragmentation, which may indicate migration limitations and disruption of the natural flow regime	Jouffray et al. 2015
238	Former pineapple/sugarcane plantation index	Landuse	Disturbance (Anthropogenic)	Proxy	Representation of lands that were formerly pineapple/sugarcane plantations that may contribute to disturbance through legacy effects	Jouffray et al. 2015
239	Ditch index	Ditch infrastructure	Disturbance (Anthropogenic)	Proxy	Relative density of ditch infrastructure.	Jouffray et al. 2015
240	Agricultural index	Agricultural disturbance	Disturbance (Anthropogenic)	Proxy	Representation of agricultural disturbance within a catchment.	Jouffray et al. 2015
241	Reef zone	Reef zone	Location & Geomorphology	State		Jouffray et al. 2015
242	Hard coral cover	Coral cover	Coral cover	State	Used in PCA to cluster into 3 regimes	Jouffray et al. 2015
243	Macroalgae cover	Macroalgae	Algal cover	State	Used in PCA to cluster into 3 regimes	Jouffray et al. 2015
244	Turf algae cover	Turf	Algal cover	State	Used in PCA to cluster into 3 regimes	Jouffray et al. 2015
245	CCA cover	CCA	Algal cover	State	Used in PCA to cluster into 3 regimes	Jouffray et al. 2015
246	Structural complexity	Rugosity	Location & Geomorphology	State/Proxy	Used in PCA to cluster into 3 regimes, and a key aspect of reef habitat quality	Jouffray et al. 2015
247	Sand cover	Substrate	Substrate	State	Used in PCA to cluster into 3 regimes	Jouffray et al. 2015

248	Connectivity	Connectivity	Connectivity	Process	Reef recovery depends on nearby and upstream reefs to provide recruits	Maynard et al. 2010
249	Free from water pollution	Pollution	Disturbance (Anthropogenic)	State/Proxy	Pesticides, nutrients and pathogens increase susceptibility of corals to disease and bleaching, increase algal growth rates	Maynard et al. 2010
250	Previous exposure to thermal stress events	Thermal regime	Physical characteristics	State/Proxy	Previous exposure, tolerance/quick recovery from thermal stress events suggests same can occur in future	Maynard et al. 2010
251	Coral cover	Coral cover	Coral cover	State/Proxy	Recruitment and intensifies grazing in areas available for algal colonisation	Maynard et al. 2010
252	Abundance of resistant/tolerant species	Resistant corals	Coral community	State/Proxy	Massive corals more tolerant of thermal and physical stress	Maynard et al. 2010
253	Water mixing	Mixing	Physical characteristics	Process	Mixing keeps water temperatures constant and reduces chances of corals in shallow water from heating	Maynard et al. 2010
254	Free from physical impacts	Physical impact	Disturbance (Physical)	State/Proxy	Wave action produced by storms can cause breakage of colonies, increase susceptibility to disease and bleaching	Maynard et al. 2010
255	Abundance of mature coral colonies	Mature coral colonies	Coral community	State/Proxy	Large colonies survived long and withstood range of stressors, also likely to produce more recruits	Maynard et al. 2010
256	Substrate availability	Substrate availability	Substrate	State/Proxy	Recruits need suitable substrate to settle on and grow	Maynard et al. 2010
257	Free from anthropogenic physical impacts	Human impact	Disturbance (Anthropogenic)	Proxy	Anchors, fins of snorkelers and divers can damage corals and increase susceptibility to disease and bleaching	Maynard et al. 2010
258	Herbivore abundance	Herbivore abundance	Herbivory	State/Proxy	Reduce cover of algae that compete with corals for space	Maynard et al. 2010
259	Exposure to upwelling	Upwelling	Physical characteristics	Process	Cool water reduces temperature stress and alleviate reefs from mass bleaching events	Maynard et al. 2010
260	Light reduction	Shading	Physical characteristics	Proxy	Reduce thermal stress to coral communities	Maynard et al. 2010
261	Free from fishing pressure	Fishing pressure	Disturbance (Anthropogenic)	Proxy	Fish important for grazing, particularly post-disturbance to free up space for coral recruits	Maynard et al. 2010
262	Topographic complexity	Rugosity	Location &	State/Proxy	Self-shading can reduce light stress that can cause bleaching	Maynard et al. 2010

			Geomorphology			
263	Coral submersion	Reef exposure at low tide	Location & Geomorphology	Proxy	Stress to corals exposed to air for long periods can cause mortality	Maynard et al. 2010
264	Free from sedimentation	Sedimentation	Disturbance (Anthropogenic)	State/Proxy	Tolerating sediment requires physiological resources increasing susceptibility to corals to other stressors	Maynard et al. 2010
265	Absence of bioeroders	Bioeroder abundance	Disturbance (Biological)	State/Proxy	At high abundances can reduce integrity of reef framework, increases susceptibility to physical stresses such as waves caused by storms	Maynard et al. 2010
266	Absence of coral disease	Disease	Disturbance (Physiological)	State/Proxy	Disease can quickly and kill colonies, and increase susceptibility of corals to other stressors	Maynard et al. 2010
267	Resistant coral species	Resistant corals	Coral community	State/Proxy	Confers resistance	McClanahan et al. 2012
268	Temperature variability	Thermal regime	Physical characteristics	State/Proxy	Previous exposure of corals to different thermal regimes, increase resistance to bleaching	McClanahan et al. 2012
269	Nutrients (Pollution)	Nutrients	Disturbance (Anthropogenic)	State/Proxy	Nutrient pollution can reduce coral reef resistance to stress, but depends on species.	McClanahan et al. 2012
270	Sedimentation	Sedimentation	Disturbance (Anthropogenic)	State/Proxy	Increased sediment linked to decrease in thermal tolerance of corals.	McClanahan et al. 2012
271	Coral diversity	Coral diversity	Coral community	State	Diversity increases resistance but likely depend on species composition. Association unclear.	McClanahan et al. 2012
272	Herbivore biomass	Herbivore biomass	Herbivory	State/Proxy	Reduce algal competition	McClanahan et al. 2012
273	Physical human impacts	Human impact	Disturbance (Anthropogenic)	Proxy	Strong negative relationship between human impacts and corals' ability to resist stressors	McClanahan et al. 2012
274	Coral disease	Disease	Disturbance (Physiological)	State/Proxy	Corals more susceptible to bleaching after disease	McClanahan et al. 2012
275	Macroalgae cover	Macroalgae	Algal cover	State/Proxy	Macroalgae reduce coral growth rates	McClanahan et al. 2012
276	Recruitment	Recruitment	Recruitment	Process	Coral recruits and small size classes more resistant to bleaching and mortality	McClanahan et al. 2012
277	Fishing pressure	Fishing pressure	Disturbance	Proxy	Indirect impact of fishing pressure	McClanahan et al. 2012

			(Anthropogenic)			
278	Sea surface temperature	Temperature	Physical characteristics	State/Proxy		Mumby et al. 2014
279	Live coral cover	Coral cover	Coral cover	State		Mumby et al. 2014
280	Spawning coral cover	Coral cover (repro group)	Coral community	State/Proxy		Mumby et al. 2014
281	Brooding coral cover	Coral cover (repro group)	Coral community	State/Proxy		Mumby et al. 2014
282	Parrotfish biomass	Herbivore biomass	Herbivory	State/Proxy		Mumby et al. 2014
283	Wave exposure index	Wave exposure	Physical characteristics	Proxy		Mumby et al. 2014
284	Hurricane occurrence	Storm	Disturbance (Physical)	State/Proxy		Mumby et al. 2014
285	Hurricane intensity	Storm intensity	Disturbance (Physical)	State/Proxy		Mumby et al. 2014
286	Coral recruitment	Recruitment	Recruitment	Process		Mumby et al. 2014
287	Coral growth	Coral growth	Growth	Process		Mumby et al. 2014
288	Coral reproduction	Coral reproduction	Reproduction	Process		Mumby et al. 2014
289	Cropped algae	Substrate availability	Substrate	State/Proxy	Grazing results in cropped algae - corals can recruit to cropped algae includes filamentous, coralline red algae and short turfs <5mm. Proxy for substrate availability.	Mumby et al. 2014
290	Site exposure (windward or leeward)	Site exposure	Location & Geomorphology	State/Proxy		Mumby et al. 2014
291	Season	Season	Physical characteristics	State		Mumby et al. 2014
292	*Dictyota* growth	Algal growth	Growth	Process		Mumby et al. 2014

293	*Lobophora* growth	Algal growth	Growth	Process		Mumby et al. 2014
294	Competition (corals)	Competition	Competition	Process		Mumby et al. 2014
295	Competition (corals and cropped algae)	Competition	Competition	Process		Mumby et al. 2014
296	Competition (corals and macroalgae): effect of macroalgae on corals	Competition	Competition	Process		Mumby et al. 2014
297	Competition (corals and macroalgae): effect of corals on macroalgae	Competition	Competition	Process		Mumby et al. 2014
298	Grazing by fishes and impact of fishing	Herbivory	Herbivory	Process		Mumby et al. 2014
299	Partial-colony mortality of corals	Coral mortality	Mortality	Process		Mumby et al. 2014
300	Whole-colony mortality of juvenile and adult corals	Coral mortality	Mortality	Process		Mumby et al. 2014
301	Parrotfish predation on coral recruits	Corallivory	Disturbance (Biological)	Process		Mumby et al. 2014
302	Hurricane impact on juvenile & mature corals (>60 cm^2): whole-colony mortality	Coral mortality	Mortality	Process		Mumby et al. 2014
303	Hurricane impact on mature corals (>250 cm^2): partial-colony mortality	Coral mortality	Mortality	Process		Mumby et al. 2014
304	Hurricane impact on juvenile corals (1-60 cm^2)	Coral mortality	Mortality	Process		Mumby et al. 2014

305	Hurricane impact on macroalgae	Algal mortality	Mortality	Process		Mumby et al. 2014
306	Bleaching impact on corals: whole-colony mortality	Coral mortality	Mortality	Process		Mumby et al. 2014
307	Bleaching impact on corals: partial-colony mortality	Coral mortality	Mortality	Process		Mumby et al. 2014
308	Bleaching frequency	Bleaching	Disturbance (Physiological)	State/Proxy		Mumby et al. 2014
309	Degree heating weeks	Thermal regime	Physical characteristics	State/Proxy		Mumby et al. 2014
310	Ungrazable substratum	Substrate	Herbivory	Proxy		Mumby et al. 2014
311	Macroalgae cover	Macroalgae	Algal cover	State/Proxy	Used to indicate coral-algal balance.	Mumby et al. 2014
312	Turf cover	Turf	Algal cover	State		Mumby et al. 2014
313	Wind direction	Wind	Physical characteristics	State/Proxy	Used to derive wind exposure	Mumby et al. 2014
314	Wind strength	Wind	Physical characteristics	State/Proxy	Used to derive wind exposure	Mumby et al. 2014
315	Fetch	Fetch	Physical characteristics	State/Proxy	Used to derive wind exposure	Mumby et al. 2014
316	Rhodolith algae	CCA	Algal cover	State	Rhodolith belongs under CCA category	NOAA CCMA-BB
317	Crustose coralline algae	CCA	Algal cover	State		NOAA CCMA-BB
318	Cyanobacteria	Cyanobacteria	Algal cover	State		NOAA CCMA-BB
319	Filamentous algae	Turf	Algal cover	State	Categorized as turf	NOAA CCMA-BB
320	Macroalgae	Macroalgae	Algal cover	State		NOAA CCMA-BB

321	Turf algae	Turf	Algal cover	State		NOAA CCMA-BB
322	Macroalgal canopy height	Algal height	Algal height	State/Proxy		NOAA CCMA-BB
323	Abiotic - Hard bottom	Substrate	Substrate	State		NOAA CCMA-BB
324	Abiotic - Sand	Substrate	Substrate	State		NOAA CCMA-BB
325	Abiotic - Rubble	Substrate	Substrate	State		NOAA CCMA-BB
326	Abiotic - Fine sediment	Substrate	Substrate	State		NOAA CCMA-BB
327	Abiotic - Rugosity	Rugosity	Location & Geomorphology	State/Proxy		NOAA CCMA-BB
328	Abiotic - Water Depth	Depth	Location & Geomorphology	State		NOAA CCMA-BB
329	Coral cover (Species)	Coral cover (species)	Coral cover	State		NOAA CCMA-BB
330	Coral diversity	Coral diversity	Coral community	State		NOAA CCMA-BB
331	Bleached coral cover	Bleached coral cover	Disturbance (Physiological)	State/Proxy		NOAA CCMA-BB
332	Recently dead coral cover	Dead coral cover	Mortality	State/Proxy		NOAA CCMA-BB
333	Seagrass cover	Seagrass	Seagrass	State		NOAA CCMA-BB
334	Seagrass height	Seagrass	Seagrass	State		NOAA CCMA-BB
335	Gorgonian cover	Other Invertebrates (Sessile)	Other invertebrates	State		NOAA CCMA-BB
336	Gorgonian height	Other Invertebrates (Sessile)	Other invertebrates	State		NOAA CCMA-BB
337	Gorgonian abundance	Other Invertebrates	Other invertebrates	State		NOAA CCMA-BB

		(Sessile)				
338	Sponge abundance	Other Invertebrates (Sessile)	Other invertebrates	State		NOAA CCMA-BB
339	Sponge cover	Other Invertebrates (Sessile)	Other invertebrates	State		NOAA CCMA-BB
340	Sponge canopy height	Other Invertebrates (Sessile)	Other invertebrates	State		NOAA CCMA-BB
341	Other benthic - Anemonies & hydroids cover	Other Invertebrates (Sessile)	Other invertebrates	State		NOAA CCMA-BB
342	Other benthic - Anemonies & hydroids abundance	Other Invertebrates (Sessile)	Other invertebrates	State		NOAA CCMA-BB
343	Other benthic - Tunicates & zoanthids	Other Invertebrates (Sessile)	Other invertebrates	State		NOAA CCMA-BB
344	Macro-invertebrates - queen conch abundance & maturity	Motile invertebrates	Other invertebrates	State		NOAA CCMA-BB
345	Macro-invertebrates - spiny lobster abundance	Motile invertebrates	Other invertebrates	State		NOAA CCMA-BB
346	Macro-invertebrates - long-spined sea urchin	Urchin	Other invertebrates	State/Proxy		NOAA CCMA-BB
347	Marine debris	Garbage	Disturbance (Anthropogenic)	State/Proxy	E.g. cloth, champagne bottle, plastic cylinder, rectangular frame and glass bottles etc.	NOAA CCMA-BB
348	Fish abundance (species)	Fish abundance (species)	Fish abundance	State		NOAA CCMA-BB
349	Fish diversity	Fish diversity	Fish community	State		NOAA CCMA-BB
350	Fish size class	Fish size	Fish size	State		NOAA CCMA-BB

351	Fish biomass (estimated)	Fish biomass	Fish biomass	State/Proxy		NOAA CCMA-BB
352	Habitat	Habitat	Location & Geomorphology	State	Hard/soft or mangrove	NOAA CCMA-BB
353	Season	Season	Physical characteristics	State		NOAA CCMA-BB
354	Herbivore biomass	Herbivore biomass	Herbivory	State/Proxy		NOAA CCMA-BB
355	Parrotfish biomass	Herbivore biomass	Herbivory	State/Proxy		NOAA CCMA-BB
356	Temperature	Temperature	Physical characteristics	State/Proxy		NOAA CCMA-BB
357	Conductivity	Conductivity	Physical characteristics	State		NOAA CCMA-BB
358	Turbidity	Turbidity	Physical characteristics	State/Proxy		NOAA CCMA-BB
359	Chlorophyll-a	Chlorophyll	Disturbance (Anthropogenic)	State/Proxy		NOAA CCMA-BB
360	Rugosity	Rugosity	Location & Geomorphology	State/Proxy		NOAA CCMA-BB
361	Hard coral cover	Coral cover	Coral cover	State/Proxy	Primary indicator of reef health	IUCN
362	Soft coral cover	Other Invertebrates (Sessile)	Other invertebrates	State/Proxy	Competitor, indicate nutrient and wave energy conditions	IUCN
363	Macroalgal Cover	Macroalgae	Algal cover	State/Proxy	Competitor, indicate nutrient/bottom-up/herbivory/top-down processes	IUCN
364	Turf Algae Cover	Turf	Algal cover	State/Proxy	Competitor, indicate nutrient/bottom-up/herbivory/top-down processes	IUCN
365	CCA Cover	CCA	Algal cover	State/Proxy	Indicator for suitable habitat for coral recruitment, consolidation of reef framework	IUCN
366	Rubble	Substrate	Substrate	State/Proxy	Indicator of substratum integrity, suitability for coral recruitment and growth	IUCN

367	Substrate & Morphology (Topographic complexity - micro)	Rugosity	Location & Geomorphology	State/Proxy	Relevant to the recruitment of corals.	IUCN
368	Substrate & Morphology (Topographic complexity - macro)	Rugosity	Location & Geomorphology	State/Proxy	Relevant as habitat for bigger organisms	IUCN
369	Substrate & Morphology (Sediment texture)	Sediment size	Physical characteristics	State/Proxy	Sediment grain size and sorting affects benthic organisms	IUCN
370	Substrate & Morphology (Sediment layer)	Sediment abundance	Physical characteristics	State/Proxy	Depth of sediment layer on hard substrata, particularly in association with algal filaments/turf	IUCN
371	Cooling & Flushing (Temperature)	Temperature	Physical characteristics	State/Proxy	Primary stressor for bleaching related to climate change	IUCN
372	Cooling & Flushing (Currents)	Current	Physical characteristics	Proxy	Currents cause vertical mixing that may reduce surface temperature	IUCN
373	Cooling & Flushing (Wave energy/exposure)	Wave exposure	Physical characteristics	Proxy	Wave energy causes vertical mixing, also represents exposure to weather events	IUCN
374	Cooling & Flushing (Proximity to deep water (30-50m))	Upwelling	Physical characteristics	State/Proxy	Proximity to deep water enables mixing with cold water by upwelling and waves, currents and exposure. Changed from deep water to upwelling since meant for it to be a proxy of upwelling.	IUCN
375	Cooling & Flushing (Depth of reef base)	Mixing	Physical characteristics	State/Proxy	Depth of base of reef slope affects potential mixing of deep cool waters. Changed from depth of reef base to mixing because proxy for mixing	IUCN
376	Shade & Screen (Depth)	Depth	Location & Geomorphology	State/Proxy	Zonation for reef and community structure, attenuation of temp, light and other variables	IUCN
377	Shade & Screen (Visibility (m))	Turbidity	Physical characteristics	State/Proxy	Proxy for turbidity and attenuation of light levels	IUCN
378	Shade & Screen (Compass direction/aspect)	Aspect	Physical characteristics	State/Proxy	Aspect of a reef slope affects the angle of incidence of the sun of reef surface (radiation per area of reef)	IUCN
379	Shade & Screen (Slope	Slope	Location &	State/Proxy	Angle of reef slope affects the angle of incidence of the sun	IUCN

	(degrees))		Geomorphology			
380	Shade & Screen (Physical shading)	Shading	Physical characteristics	Proxy	Shading can protect corals from stress	IUCN
381	Shade & Screen (Canopy corals)	Coral cover (growth form)	Coral community	State/Proxy	Shading of understory corals by canopy corals (table, staghorn, plates) can protect them from stress	IUCN
382	Extremes & Acclimatization (Temperature variability)	Thermal regime	Physical characteristics	State/Proxy	Variability promotes acclimatization over time, but only up to a certain point, suggests to interpret with local knowledge	IUCN
383	Extremes & Acclimatization (Exposed low tide)	Reef exposure at low tide	Location & Geomorphology	Proxy	Corals exposed to air experience frequent stress and may be more resistant to thermal stress	IUCN
384	Extremes & Acclimatization (Ponding/pooling)	Ponding/pooling	Physical characteristics	State/Proxy	Restricted bodies of water heat up more and are more stressed	IUCN
385	Extremes & Acclimatization (Survival of past bleaching events)	Thermal regime	Physical characteristics	State/Proxy	Evidence of corals been bleached and survived, survival indicates acclimatization	IUCN
386	Bleaching	Bleaching	Disturbance (Physiological)	State/Proxy	Current levels of bleaching	IUCN
387	Mortality-recent	Dead coral cover	Mortality	State/Proxy	Current levels of mortality - partial/full mortality	IUCN
388	Coral disease	Disease	Disturbance (Physiological)	State/Proxy	Percent corals showing disease conditions	IUCN
389	Mortality-old	Dead coral cover	Coral cover	State		IUCN
390	Recovery-old	Dead coral cover	Coral cover	State		IUCN
391	Coral population structure	Coral population structure	Coral community	Proxy	Represents succession of coral community as documented by the next 4 variables.	IUCN
392	Population Biology	Juvenile corals	Recruitment	State/Proxy	Estimated number of recruits <2-3cm per square meter	IUCN

	(Recruitment)					
393	Population Biology (Fragmentation)	Fragmentation	Reproduction	State/Proxy	Estimated contribution of fragmentation in producing new colonies from 5-20cm	IUCN
394	Population Biology (Dominant size class)	Coral size	Coral community	State/Proxy	Estimate dominance in coral community by size class, indicating successional stage of community	IUCN
395	Population Biology (Largest corals)	Coral size	Coral community	State/Proxy	Largest corals at a site indicate suitability of site and degree of env. Stability/community persistence	IUCN
396	Positive coral associates (Obligate feeders)	Coral-dependent organisms	Organisms (Unharmful)	State/Proxy	Abundance and diversity of obligate coral feeders indicative of health of coral colonies and complexity of interactions	IUCN
397	Positive coral associates (Branching residents (Abundance and diversity of fish/crustacean residents in corals))	Coral-dependent organisms	Organisms (Unharmful)	State/Proxy	Abundance and diversity of fish and invertebrate residents are indicative of health and complexity of interactions	IUCN
398	Negative coral associates (Competitors)	Competitor abundance	Competition	State/Proxy	Abundance and diversity of coral competitors indicative of inhibiting factors to coral growth and recovery	IUCN
399	Negative coral associates (Bioeroders (internal))	Bioeroder abundance	Disturbance (Biological)	State/Proxy	Urchins, nonfish - inhibiting factors to coral growth and recovery	IUCN
400	Negative coral associates (Bioeroders (external))	Bioeroder abundance	Disturbance (Biological)	State/Proxy	Sponges, worms - inhibiting factors to coral growth and recovery	IUCN
401	Negative coral associates (Corallivores (negative))	Corallivores	Disturbance (Biological)	State/Proxy	Indicative to additional mortality to coral colonies	IUCN
402	Herbivore diversity	Herbivore diversity	Fish community	State/Proxy	Overall critical for suppressing algal growth and inhibiting effects on corals	IUCN
403	Herbivore abundance (Excavators)	Herbivore abundance	Herbivory	State/Proxy	Control on algal growth	IUCN
404	Herbivore abundance (Scrapers)	Herbivore abundance	Herbivory	State/Proxy	Control on macroalgal fronds	IUCN
405	Herbivore abundance	Herbivore	Herbivory	State/Proxy	Control on epilithic turf algae	IUCN

	(Grazers/browsers)	abundance				
406	Top Predators (Piscivores)	Fish abundance (key)	Fish abundance	State		IUCN
407	Dispersal (self-seeding)	Larval supply	Connectivity	Process	Recruitment of new corals driven more by self-seeding	IUCN
408	Dispersal (local-seeding)	Larval supply	Connectivity	Process	Larval density decreases with distance from source	IUCN
409	Dispersal (distant-seeding)	Larval supply	Connectivity	Process	Larval density decreases with distance from source	IUCN
410	Transport (Currents)	Current	Connectivity	Proxy		IUCN
411	Transport (Dispersal barrier)	Dispersal barrier	Location & Geomorphology	State/Proxy	Barriers reduce larvae flow	IUCN
412	Water (Nutrient input)	Nutrients	Disturbance (Anthropogenic)	State/Proxy	Nutrient enhancement alter reef processes	IUCN
413	Water (Pollution (Chemical))	Pollution	Disturbance (Anthropogenic)	State/Proxy	Reduce ability to withstand other stresses	IUCN
414	Substrate (Pollution (Solid))	Garbage	Disturbance (Anthropogenic)	State/Proxy	Unsuitable for coral growth and recruitment	IUCN
415	Turbidity/sedimentation	Turbidity	Physical characteristics	State/Proxy	Anthropogenic enhanced turbidity and sedimentation negatively affects corals	IUCN
416	Anthropogenic (Physical damage)	Human impact	Disturbance (Anthropogenic)	Proxy	Mortality/inhibits recovery	IUCN
417	Fishing pressure	Fishing pressure	Disturbance (Anthropogenic)	Proxy	Overfishing causes reef degradation	IUCN
418	Destructive fishing	Destructive fishing	Disturbance (Physical)	State/Proxy	Physical damage to site, alters balance of fish dynamics	IUCN
419	Connectivity (Dispersal barrier) Anthropogenic	Dispersal barrier	Disturbance (Anthropogenic)	State/Proxy	Construction that affects connectivity	IUCN

420	Management (Biodiversity)	Management	Management Status	State/Proxy	Protection of biodiversity	IUCN
421	Management (Environmental quality)	Management	Management Status	State/Proxy	Protection from extraction of resources by fishing or other activities	IUCN
422	Management (Resources)	Management	Management Status	State/Proxy	Limitation of human activities that degrade the environment, e.g. pollution	IUCN
423	Depth	Depth	Location & Geomorphology	State		Reef Check
424	Latitude and longitude	Latitude/Longitude	Location & Geomorphology	State		Reef Check
425	Site exposure (windward or leeward)	Site exposure	Location & Geomorphology	State/Proxy		Reef Check
426	Impacts - Blast/poison/net aquarium fishing/ collecting invertebrates for food/curio sales/tourists	Human impact	Disturbance (Anthropogenic)	Proxy		Reef Check
427	Impacts - Sewage/industrial pollution	Pollution	Disturbance (Anthropogenic)	State/Proxy		Reef Check
428	Impacts - Commercial fishing	Fishing pressure	Disturbance (Anthropogenic)	Proxy		Reef Check
429	Number of yachts/motorboats	Human impact	Disturbance (Anthropogenic)	Proxy		Reef Check
430	Management	Management	Management Status	State/Proxy		Reef Check
431	Fish abundance (Selected species)	Fish abundance (key)	Fish abundance	State		Reef Check
432	Fish size class distribution (Selected	Fish size (key)	Fish size	State		Reef Check

	species)					
433	Urchin	Urchin	Herbivory	State/Proxy	Important algae grazers	Reef Check
434	COTS abundance	COTS	Disturbance (Biological)	State/Proxy	Coral damage	Reef Check
435	Triton abundance	Motile invertebrates	Other invertebrates	State		Reef Check
436	Banded coral shrimp	Motile invertebrates	Other invertebrates	State		Reef Check
437	Lobster abundance	Motile invertebrates	Other invertebrates	State/Proxy	Usually seen only on reefs that have not been overfished	Reef Check
438	Giant clam abundance	Motile invertebrates	Other invertebrates	State/Proxy	Usually seen only on reefs that have not been overfished	Reef Check
439	Triton abundance	Motile invertebrates	Other invertebrates	State/Proxy	Usually seen only on reefs that have not been overfished	Reef Check
440	Gorgonian	Other Invertebrates (Sessile)	Other invertebrates	State		Reef Check
441	Cowries abundance	Motile invertebrates	Other invertebrates	State		Reef Check
442	Coral disease	Disease	Disturbance (Physiological)	State/Proxy		Reef Check
443	Bleached coral cover	Bleached coral cover	Disturbance (Physiological)	State/Proxy		Reef Check
444	Coral cover (species)	Coral cover (species)	Coral cover	State		Reef Check
445	Coral diversity	Coral diversity	Coral community	State		Reef Check
446	Soft coral cover	Other Invertebrates (Sessile)	Other invertebrates	State		Reef Check

447	Recently killed coral	Dead coral cover	Mortality	State/Proxy		Reef Check
448	Nutrient indicator algae	Macroalgae	Algal cover	State/Proxy	Macroalgae that indicates nutrient presence. All algae except coralline, calcareous (*Halimeda*) and turf	Reef Check
449	Turf algae	Turf	Algal cover	State		Reef Check
450	CCA algae	CCA	Algal cover	State		Reef Check
451	Sponge	Other Invertebrates (Sessile)	Other invertebrates	State		Reef Check
452	Rock	Substrate	Substrate	State		Reef Check
453	Rubble	Substrate	Substrate	State		Reef Check
454	Sand	Substrate	Substrate	State		Reef Check
455	Silt/Clay	Substrate	Substrate	State		Reef Check
456	Other organisms (sea anemones, tunicates, gorgonians or non-living substrate)	Other Invertebrates (Sessile)	Other invertebrates	State		Reef Check
457	Physical damage	Physical impact	Disturbance (Physical)	State/Proxy		Reef Check
458	Reef zone	Reef zone	Location & Geomorphology	State		Reef Check
459	Habitat	Habitat	Location & Geomorphology	State		Reef Check
460	Live coral abundance	Coral cover	Coral cover	State/Proxy	Indicative of past stress tolerance; more corals will produce more gametes. Used total coral cover, not coral size, coral genus in S2 because it is only used to prove that depth should be included in the RSRI.	Rowlands et al. 2012
461	Habitat	Habitat	Location & Geomorphology	State		Rowlands et al. 2012

462	Framework abundance index	Substrate availability	Substrate	Proxy	Proportion of grid cell occupied by coral framework (alive/dead) suitable for coral recruitment. When reef framework is abundant close to source patch, more likely to be colonized.	Rowlands et al. 2012
463	Sand, rubble, sediment	Substrate	Substrate	State		Rowlands et al. 2012
464	Macroalgae	Macroalgae	Algal cover	State		Rowlands et al. 2012
465	Water depth variability	Depth variability	Physical characteristics	State/Proxy	Species show different stress tolerance, cooler regime found in deeper areas yielding a thermal stress refuge. Ratio of SD of water depth at site to maximum SD of water depth for 5 study sites	Rowlands et al. 2012
466	Water depth	Depth	Location & Geomorphology	State		Rowlands et al. 2012
467	Fishing intensity	Fishing pressure	Disturbance (Anthropogenic)	Proxy	Removal of herbivorous fish affects coral-algal balance; removal of predators alters dynamics and food web structure. Combine industrial and traditional fishing pressure	Rowlands et al. 2012
468	Industry and development	Development index	Disturbance (Anthropogenic)	Proxy	Light proximity index, with assumption that reefs are more impacted if close to light source at night. Nutrient enrichment, pollution, desalination outflow, oil cause stress, reduced productivity or death. Development (industrial and residential) have harmful impacts to corals across range of distances from source, typically most harmful to close proximity	Rowlands et al. 2012
469	Thermal stress index	Thermal regime	Disturbance (Physiological)	Proxy	Warming waters cause stress, reduced productivity and ultimately death through coral bleaching	Rowlands et al. 2012
470	Sea surface temperature	Temperature	Physical characteristics	State/Proxy	To calculate thermal stress index	Rowlands et al. 2012
471	Degree heating days	Thermal regime	Physical characteristics	State/Proxy	To calculate thermal stress index, considered a measure of chronic thermal stress, the slow exposure of corals to abnormally high temperature	Rowlands et al. 2012
472	Heating rate	Heating rate	Physical characteristics	Process	To calculate thermal stress index	Rowlands et al. 2012
473	High recovery rate from past disturbances	Recovery rate	Coral community	Process	Past recovery an indicator of future recovery	Weeks & Jupiter 2013
474	Coral population	Coral population	Coral community	State/Proxy	Proxy (juvenile coral density). High abundance of mature colonies + strong	Weeks & Jupiter 2013

	structure	structure			recruitment an indicator of strong future recovery	
475	Juvenile coral density	Juvenile corals	Recruitment	State/Proxy	Evidence of strong coral recruitment are likely to demonstrate high potential to withstand and recover from disturbance	Weeks & Jupiter 2013
476	Connectivity	Connectivity	Connectivity	Process	Proxy: Juvenile coral density. Protected sites well connected to each other may provide larvae to promote recovery	Weeks & Jupiter 2013
477	Substrate availability	Substrate availability	Substrate	State/Proxy	High availability of suitable substrate on which coral recruits can settle likely to promote recovery	Weeks & Jupiter 2013
478	Herbivore abundance	Herbivore abundance	Herbivory	State/Proxy	Proxy: Herbivore biomass. High abundance of herbivores likely to reduce harmful coral-algal interactions and provide substrate for coral recruits to settle	Weeks & Jupiter 2013
479	Herbivore functional diversity	Herbivore diversity	Fish community	State/Proxy	Measure: Pielou's evenness of herbivore functional groups present. High functional diversity of herbivores likely to reduce harmful coral-algal interactions and provide substrate for coral recruits to settle	Weeks & Jupiter 2013
480	Abundance of bioeroders	Bioeroder abundance	Disturbance (Biological)	State/Proxy	Reefs with low abundance of bioeroders are more resistant to other forms of disturbance	Weeks & Jupiter 2013
481	Absense of coral disease	Disease	Disturbance (Physiological)	State/Proxy	Low disease likely to be more resistant to other forms of disturbance	Weeks & Jupiter 2013
482	Fish diversity	Fish diversity	Fish community	State/Proxy	High fish diversity & functional diversity likely to demonstrate higher resilience	Weeks & Jupiter 2013
483	Fish size	Fish size	Fish size	State		Weeks & Jupiter 2013
484	Fish abundance	Total fish abundance	Fish abundance	State		Weeks & Jupiter 2013
485	Fish biomass (estimated)	Fish biomass	Fish biomass	State/Proxy		Weeks & Jupiter 2013
486	Coral diversity	Coral diversity	Coral community	State	High coral diversity likely to demonstrate higher resilience	Weeks & Jupiter 2013
487	Coral cover	Coral cover	Coral cover	State		Weeks & Jupiter 2013
488	Coral community dominated by resistant/tolerant taxa	Resistant corals	Coral community	State/Proxy	More resistant to disturbance	Weeks & Jupiter 2013

489	Fishing pressure	Fishing pressure	Disturbance (Anthropogenic)	Proxy	Low fishing pressure/not targetting herbivores likely to show higher resilience	Weeks & Jupiter 2013
490	Anthropogenic physical impacts	Human impact	Disturbance (Anthropogenic)	Proxy	Reefs not subjective to pressure are likely to have higher resilience	Weeks & Jupiter 2013
491	Water quality	Water quality	Disturbance (Anthropogenic)	State/Proxy	Water pollution and unnatural levels of nutrients impact resistance and recovery	Weeks & Jupiter 2013
492	Reef aspect	Aspect	Physical characteristics	State/Proxy	Sites angled away from sunlight may be less exposed to thermal stress	Weeks & Jupiter 2013
493	Depth	Depth	Location & Geomorphology	State/Proxy	Deeper sites cooler, but shallow sites more acclimated to hot temperatures	Weeks & Jupiter 2013
494	Reef exposure at low tide	Reef exposure at low tide	Location & Geomorphology	Proxy	Proxy: Reef exposure at low tide. Reefs routinely exposed are better adapted to thermal stress, but extremely low tides with high noon irradiances may result in bleaching mortality	Weeks & Jupiter 2013
495	Proximity to upwelling or exposure to strong currents	Upwelling	Physical characteristics	State/Proxy	Sites influenced by upwelling may not experience as severe positive temperature anomalies	Weeks & Jupiter 2013
496	Shading from high, steep sided islands or reef topography	Shading	Physical characteristics	Proxy	Shaded sites may not experience as severe positive temperature anomalies	Weeks & Jupiter 2013
497	Turbid water	Turbidity	Physical characteristics	State/Proxy		Weeks & Jupiter 2013
498	Natural physical impacts	Physical impact	Disturbance (Physical)	State/Proxy	Sites not frequently physically disturbed likely to show higher resilience	Weeks & Jupiter 2013
499	Resistance (physical factors that reduce temp. stress) - Exchange	Exchange	Physical characteristics	Process	Warm water replaced with cooler oceanic water	West & Salm 2003
500	Resistance (physical factors that reduce temp. stress) - Upwelling	Upwelling	Physical characteristics	Process		West & Salm 2003

501	Resistance (physical factors that reduce temp. stress) - Areas adjacent to deep water	Deep water	Physical characteristics	State/Proxy		West & Salm 2003
502	Resistance (physical factors that reduce temp. stress) - Wind-driven mixing	Mixing	Physical characteristics	Process		West & Salm 2003
503	Resistance (physical factors that enhance water movement and flush toxins) - Fast currents	Current	Physical characteristics	Proxy	Eddies, tidal and ocean currents, gyres	West & Salm 2003
504	Resistance (physical factors that enhance water movement and flush toxins) - Topography	Topography	Location & Geomorphology	State/Proxy	Does not refer to rugosity, but of geological structures promoting water flow	West & Salm 2003
505	Resistance (physical factors that enhance water movement and flush toxins) - High wave energy	Wave exposure	Physical characteristics	Proxy		West & Salm 2003
506	Resistance (physical factors that enhance water movement and flush toxins) - Tidal range	Tide	Physical characteristics	State/Proxy		West & Salm 2003
507	Resistance (physical factors that enhance water movement and flush toxins) - Wind	Wind	Physical characteristics	State/Proxy		West & Salm 2003
508	Resistance (physical factors that decrease light	Shading	Physical characteristics	Proxy		West & Salm 2003

	stress) - Shade					
509	Resistance (physical factors that decrease light stress) - Aspect relative to the sun	Aspect	Physical characteristics	State/Proxy		West & Salm 2003
510	Resistance (physical factors that decrease light stress) - Slope	Slope	Location & Geomorphology	State/Proxy		West & Salm 2003
511	Resistance (physical factors that decrease light stress) - Turbidity	Turbidity	Physical characteristics	State/Proxy		West & Salm 2003
512	Resistance (physical factors that decrease light stress) - Absorption/CDOM	CDOM	Disturbance (Anthropogenic)	State/Proxy		West & Salm 2003
513	Resistance (physical factors that decrease light stress) - Cloud cover	Cloud cover	Physical characteristics	State/Proxy		West & Salm 2003
514	Resistance (factors that correlate with bleaching tolerance) - Temperature variability	Thermal regime	Physical characteristics	State/Proxy		West & Salm 2003
515	Resistance (factors that correlate with bleaching tolerance) - Emergence at low tide	Reef exposure at low tide	Location & Geomorphology	Proxy		West & Salm 2003
516	Resistance (indirect indicators of bleaching tolerance) - Broad size and species distributions	Coral size	Coral community	State/Proxy		West & Salm 2003
517	Resistance (indirect indicators of bleaching tolerance) - Areas of	Coral cover	Coral cover	State/Proxy		West & Salm 2003

	greatest remaining coral cover					
518	Resistance (indirect indicators of bleaching tolerance) - History of corals surviving bleaching events	Thermal regime	Physical characteristics	State/Proxy		West & Salm 2003
519	Resilience (Intrinsic) - Availability and abundance of local larvae	Reproduction	Reproduction	Process		West & Salm 2003
520	Resilience (Intrinsic) - Recruitment success	Recruitment	Recruitment	Process		West & Salm 2003
521	Resilience (Intrinsic) - Low abundance of bioeroders, corallivores, diseases	Harmful organisms	Organisms (Harmful)	State/Proxy		West & Salm 2003
522	Resilience (Intrinsic) - Diverse well-balanced community to prepare substratum for coral settlement	Substrate availability	Substrate	State/Proxy		West & Salm 2003
523	Resilience (Extrinsic) - Good potential for recovery because of effective management regime	Management	Management Status	State/Proxy		West & Salm 2003
524	Resilience (Extrinsic) - Connectivity by currents	Current	Connectivity	Proxy		West & Salm 2003
525	Resilience (Extrinsic) - Concentration of larval supply	Larval supply	Connectivity	Process		West & Salm 2003

References

1. Atlantic and Gulf Rapid Reef Assessment (AGGRA), available from: http://www.agrra.org/
2. Australia Institute of Marine Science (AIMS) Long-term monitoring of the Great Barrier Reef (LTMP). Standard Operation Procedures 3, 9 and 10, available from: http://www.aims.gov.au/docs/research/monitoring/reef/reef-monitoring.html
3. Bruckner AW. Static measurements of the resilience of Caribbean coral populations. Rev Biol Trop. 2012;60:39-57.
4. CARICOMP. 2001. The Caribbean Coastal Marine Productivity Program (CARICOMP). Bulletin of Marine Science 69, 819-829.
5. Cinner JE, Huchery C, Darling ES, Humphries AT, Graham NAJ, Hicks CC, et al. Evaluating Social and Ecological Vulnerability of Coral Reef Fisheries to Climate Change. PLoS ONE. 2013;8(9):e74321.
6. Hawai'i Coral Reef Assessment and Monitoring Program (CRAMP), available from: http://cramp.wcc.hawaii.edu/
7. Graham NAJ, Jennings S, MacNeil MA, Mouillot D, Wilson SK. Predicting climate-driven regime shifts versus rebound potential in coral reefs. Nature. 2015;518(7537):94-7.
8. Coral Reef Evaluation and Monitoring Project (CREMP), available from: http://myfwc.com/research/habitat/coral/cremp/
9. Jouffray JB, Nystrom M, Norstrom AV, Williams ID, Wedding LM, Kittinger JN, et al. Identifying multiple coral reef regimes and their drivers across the Hawaiian archipelago. Philosophical Transactions of the Royal Society B-Biological Sciences. 2015;370(1659).
10. Maynard JA, Marshall PA, Johnson JE, Harman S. Building resilience into practical conservation: identifying local management responses to global climate change in the southern Great Barrier Reef. Coral Reefs. 2010;29(2):381-91.
11. McClanahan TR, Campbell SJ, Darling ES, Eakin CM, Heron SF, Jupiter SD, et al. Prioritizing key resilience indicators to support coral reef management in a changing climate. PLoS ONE. 2012;7(8):e42884.
12. Mumby PJ, Wolff NH, Bozec YM, Chollett I, Halloran P. Operationalizing the resilience of coral reefs in an era of climate change. Conservation Letters. 2014;4(7):1-12.
13. NOAA Center for Coastal Monitoring and Assessment Biogeographic Branch – Caribbean Coral Reef Ecosystem Monitoring Project (NOAA CCMA-BB), available from: http://ccma.nos.noaa.gov/ecosystems/coralreef/reef_fish/protocols.aspx
14. Obura D, Grimsditch G. Resilience assessment of coral reefs: assessment protocol for coral reefs, focusing on coral bleaching and thermal stress. Gland, Switzerland: IUCN; 2009.
15. Hodgson G, Hill J, Kiene W, Maun L, Mihaly J, Liebeler J, et al. Reef check instruction manual: A guide to reef check coral reef monitoring2006. 87 p.
16. Rowlands G, Purkis S, Riegl B, Metsamaa L, Bruckner A, Renaud P. Satellite imaging coral reef resilience at regional scale. A case-study from Saudi Arabia. Mar Pollut Bull. 2012;64(6):1222-37.
17. Weeks R, Jupiter SD. Adaptive Comanagement of a Marine Protected Area Network in Fiji. Conserv Biol. 2013;27(6):1234-44.
18. West JM, Salm RV. Resistance and resilience to coral bleaching: implications for coral reef conservation and management. Conserv Biol. 2003;17(4):956-67.
