# Supplementary material for: The influence of resilience-based management on coral reef monitoring: A systematic review
Source: PLoS One. 2017 Feb 10;12(2):e0172064. doi: 10.1371/journal.pone.0172064 (PMC5302802; doi:10.1371/journal.pone.0172064)
Supplement: S3 Table — (PDF) [file pone.0172064.s004.pdf]

S3A Table. Results from 2-way permutational MANOVA testing for differences between metrics used by study groups and location. Fixed factor: group and location.

Source of variation	df	SS	MS	Pseudo-F	P(perm)
Group	1	6324.8	6324.8	1.8631	0.001
Location	3	11637	3879.1	1.1427	0.184
Goal x Location	2	6560.9	3280.4	0.96635	0.534
Residual	16	37341	3394.7		

S3B Table. Results from PERMDISP test of homogeneity of dispersions in the metrics used by monitoring and resilience assessments. Fixed factor: group.

Group	n	Average	SE	Pseudo-F	P(perm)
Monitoring	6	47.12	2.1289	33.529	0.0007
Resilience	12	59.121	1.026		
