# Supplementary material for: The influence of resilience-based management on coral reef monitoring: A systematic review
Source: PLoS One. 2017 Feb 10;12(2):e0172064. doi: 10.1371/journal.pone.0172064 (PMC5302802; doi:10.1371/journal.pone.0172064)
Supplement: S4 Table — Results of SIMPER analysis for studies within the monitoring group (A), resilience group (B) and a comparison between the two groups (C). (PDF) [file pone.0172064.s005.pdf]

S4 Table. Results of SIMPER analysis for studies within the monitoring group (A), resilience group (B) and a comparison between the two groups (C).

**(A) Monitoring metrics**	**Average abundance**	**Average similarity**	**Sim/SD**	**Contribution (%)**	**Cumulative (%)**
Macroalgae	1	4.49	5.87	10.69	10.69
Bleached coral cover	0.83	2.98	1.31	7.09	17.78
Other Invertebrates (Sessile)	0.83	2.98	1.31	7.09	24.88
CCA	0.83	2.68	1.36	6.38	31.25
Substrate	0.83	2.68	1.36	6.38	37.63
Turf	0.83	2.68	1.36	6.38	44.01
Coral cover (species)	0.67	1.89	0.77	4.5	48.51
Coral diversity	0.67	1.89	0.77	4.5	53
Disease	0.67	1.89	0.77	4.5	57.5
Habitat	0.67	1.89	0.77	4.5	62
Urchin	0.67	1.89	0.77	4.5	66.49
Temperature	0.67	1.83	0.77	4.36	70.85
Depth	0.67	1.66	0.79	3.94	74.79
Dead coral cover	0.67	1.56	0.79	3.71	78.51
Cyanobacteria	0.5	0.99	0.48	2.36	80.87
Latitude/Longitude	0.5	0.87	0.48	2.07	82.94
Rugosity	0.5	0.84	0.48	1.99	84.93
Fish diversity	0.5	0.77	0.48	1.84	86.77
Turbidity	0.5	0.77	0.48	1.84	88.61
Motile invertebrates	0.5	0.74	0.48	1.77	90.37

**(B) Resilience metrics**	**Average abundance**	**Average similarity**	**Sim/SD**	**Contribution (%)**	**Cumulative (%)**
Macroalgae	0.67	1.92	0.77	8.7	8.7
Coral cover	0.67	1.82	0.81	8.26	16.96
Thermal regime	0.58	1.51	0.64	6.83	23.79
Depth	0.58	1.38	0.62	6.26	30.05
Substrate availability	0.5	1.07	0.53	4.83	34.88
Fishing pressure	0.5	0.99	0.49	4.49	39.37
Disease	0.5	0.94	0.5	4.24	43.61
Substrate	0.5	0.92	0.5	4.16	47.77
Herbivore biomass	0.42	0.77	0.39	3.48	51.25
Rugosity	0.42	0.7	0.4	3.16	54.41
Coral diversity	0.42	0.68	0.4	3.1	57.51
Upwelling	0.42	0.65	0.4	2.93	60.44
Juvenile corals	0.42	0.59	0.39	2.67	63.11
Temperature	0.42	0.55	0.4	2.47	65.58
Turf	0.42	0.53	0.4	2.41	67.99
Coral size	0.42	0.52	0.4	2.36	70.35
Human impact	0.33	0.39	0.3	1.78	72.12

Latitude/Longitude	0.33	0.37	0.3	1.69	73.81
Herbivore diversity	0.33	0.37	0.3	1.69	75.5
Reef exposure at low tide	0.33	0.33	0.31	1.48	76.98
Shading	0.33	0.33	0.31	1.48	78.46
Management	0.33	0.31	0.3	1.43	79.89
CCA	0.33	0.31	0.3	1.4	81.29
Other Invertebrates (Sessile)	0.33	0.3	0.3	1.35	82.64
Resistant corals	0.25	0.25	0.21	1.13	83.77
Nutrients	0.25	0.24	0.19	1.11	84.88
Recruitment	0.25	0.23	0.21	1.02	85.9
Pollution	0.25	0.17	0.21	0.77	86.67
Harmful organisms	0.25	0.17	0.21	0.76	87.43
Mixing	0.25	0.16	0.21	0.72	88.15
Bioeroder abundance	0.25	0.16	0.21	0.71	88.86
Herbivore abundance	0.25	0.16	0.21	0.71	89.56
Fish biomass	0.25	0.15	0.21	0.69	90.25

**(C) Monitoring & resilience metrics**	**Avg. abund, (M)**	**Avg. abund. (R)**	**Avg. dissimilarity**	**Diss/SD**	**Contribution (%)**	**Cumulative (%)**
Bleached coral cover	0.83	0.08	1.86	1.61	2.47	2.47
Coral cover (species)	0.67	0.08	1.52	1.19	2.02	4.49
Other Invertebrates (Sessile)	0.83	0.33	1.51	1.15	2.01	6.5
Urchin	0.67	0.17	1.46	1.14	1.94	8.44
CCA	0.83	0.33	1.43	1.19	1.91	10.35
Habitat	0.67	0.17	1.41	1.15	1.87	12.22
Thermal regime	0	0.58	1.36	1.08	1.81	14.03
Turf	0.83	0.42	1.33	1.07	1.77	15.8
Dead coral cover	0.67	0.25	1.31	1.13	1.75	17.55
Temperature	0.67	0.42	1.28	0.97	1.7	19.25
Coral cover	0.33	0.67	1.28	1.04	1.7	20.95
Coral diversity	0.67	0.42	1.24	0.97	1.66	22.6
Cyanobacteria	0.5	0.17	1.22	0.91	1.62	24.23
Substrate	0.83	0.5	1.2	0.95	1.6	25.83
Disease	0.67	0.5	1.19	0.93	1.59	27.41
Substrate availability	0	0.5	1.16	0.95	1.54	28.95
Rugosity	0.5	0.42	1.15	0.93	1.54	30.49
Latitude/Longitude	0.5	0.33	1.15	0.94	1.53	32.02
Fishing pressure	0.17	0.5	1.14	0.91	1.51	33.54
Depth	0.67	0.58	1.12	0.88	1.5	35.03
Fish diversity	0.5	0.25	1.1	0.95	1.46	36.49
Turbidity	0.5	0.25	1.1	0.96	1.46	37.95
Juvenile corals	0.33	0.42	1.06	0.88	1.41	39.36
Herbivore biomass	0.17	0.42	1.06	0.82	1.4	40.76

Motile invertebrates	0.5	0.08	1.05	0.96	1.39	42.16
Management	0.33	0.33	0.99	0.83	1.32	43.47
Upwelling	0	0.42	0.93	0.79	1.24	44.71
Fish biomass	0.33	0.25	0.9	0.81	1.2	45.91
Human impact	0.17	0.33	0.88	0.74	1.17	47.09
Fish size	0.33	0.17	0.86	0.77	1.14	48.23
Coralline algae	0.33	0.17	0.85	0.76	1.13	49.36
Seagrass	0.33	0	0.85	0.65	1.13	50.5
Fish abundance (key)	0.33	0.17	0.83	0.76	1.11	51.61
Coral size	0	0.42	0.82	0.8	1.1	52.71
Harmful organisms	0.17	0.25	0.81	0.64	1.08	53.79
Nutrients	0.17	0.25	0.81	0.65	1.08	54.86
Macroalgae	1	0.67	0.81	0.68	1.07	55.94
Total fish abundance	0.33	0.08	0.79	0.72	1.05	56.99
Chlorophyll	0.33	0.08	0.75	0.73	1	57.99
Fish size (key)	0.33	0.08	0.75	0.72	0.99	58.98
Herbivore diversity	0	0.33	0.73	0.66	0.98	59.96
Carbon	0.33	0	0.73	0.68	0.97	60.93
Nitrogen	0.33	0	0.73	0.68	0.97	61.89
Salinity	0.33	0	0.73	0.68	0.97	62.86
Pollution	0.17	0.25	0.72	0.67	0.96	63.82
Wave exposure	0.17	0.25	0.7	0.67	0.93	64.75
COTS	0.33	0	0.7	0.68	0.93	65.68
Reef exposure at low tide	0	0.33	0.68	0.68	0.9	66.58
Shading	0	0.33	0.68	0.68	0.9	67.48
Resistant corals	0	0.25	0.63	0.55	0.84	68.32
Physical impact	0.17	0.17	0.62	0.6	0.83	69.14
Recruitment	0	0.25	0.6	0.55	0.8	69.95
Tide	0.17	0.17	0.59	0.6	0.79	70.74
Reef zone	0.17	0.17	0.58	0.59	0.78	71.52
Wind	0.17	0.17	0.58	0.6	0.78	72.29
Site exposure	0.17	0.17	0.57	0.6	0.75	73.05
Garbage	0.17	0.17	0.51	0.6	0.68	73.73
Coral mortality	0.17	0.08	0.5	0.52	0.67	74.4
Mixing	0	0.25	0.5	0.55	0.67	75.07
Bioeroder abundance	0	0.25	0.5	0.55	0.66	75.73
Herbivore abundance	0	0.25	0.5	0.55	0.66	76.39
Current	0	0.25	0.48	0.56	0.64	77.03
Bleaching	0	0.25	0.48	0.56	0.64	77.67
Aspect	0	0.25	0.47	0.56	0.63	78.3
Coral cover (growth form)	0.17	0.08	0.47	0.51	0.62	78.92
Sediment size	0.17	0.08	0.47	0.51	0.62	79.55
Cloud cover	0.17	0.08	0.46	0.52	0.62	80.16
Season	0.17	0.08	0.46	0.52	0.61	80.78

Storm	0.17	0.08	0.46	0.52	0.61	81.39
Sedimentation	0	0.17	0.46	0.43	0.61	82
Algal height	0.17	0.08	0.43	0.52	0.57	82.57
Coral cover (genus)	0.17	0.08	0.43	0.52	0.57	83.13
Slope	0	0.25	0.43	0.56	0.57	83.7
Algal biomass	0.17	0	0.39	0.43	0.52	84.22
Fish size (species)	0.17	0	0.39	0.43	0.52	84.73
Connectivity	0	0.17	0.38	0.44	0.5	85.23
Water quality	0	0.17	0.36	0.44	0.48	85.71
Conductivity	0.17	0	0.34	0.43	0.45	86.16
Feeding scars	0.17	0	0.34	0.43	0.45	86.61
Fish abundance (species)	0.17	0	0.34	0.43	0.45	87.06
Larval supply	0	0.17	0.3	0.43	0.4	87.46
Coral population structure	0	0.17	0.29	0.43	0.39	87.85
Coral cover (branching)	0	0.08	0.25	0.29	0.34	88.19
Depth variability	0	0.08	0.25	0.29	0.33	88.52
Development index	0	0.08	0.25	0.29	0.33	88.85
Heating rate	0	0.08	0.25	0.29	0.33	89.17
Cause of mortality	0	0.08	0.21	0.3	0.28	89.46
Settlement location	0	0.08	0.21	0.3	0.28	89.74
Mature coral colonies	0	0.08	0.2	0.3	0.27	90.01
